# The hidden burden of eating disorders: an extension of estimates from the Global Burden of Disease Study 2019

**DOI:** 10.1016/S2215-0366(21)00040-7

**Published:** 2021-04

**Authors:** Damian F Santomauro, Sarah Melen, Deborah Mitchison, Theo Vos, Harvey Whiteford, Alize J Ferrari

**Affiliations:** a School of Public Health, The University of Queensland, Herston, QLD, Australia; b Queensland Centre for Mental Health Research, Wacol, QLD, Australia; c Institute for Health Metrics and Evaluation, University of Washington, Seattle, WA, USA; d Translational Health Research Institute, School of Medicine, Western Sydney University, Penrith, NSW, Australia; e Department of Psychology, Macquarie University, North Ryde, NSW, Australia

## Abstract

**Background:**

Anorexia nervosa and bulimia nervosa are the only eating disorders included in the Global Burden of Diseases, Injuries, and Risk Factors Study (GBD) 2019, yet binge-eating disorder and other specified feeding or eating disorder (OSFED) are more prevalent. This study sought to estimate the prevalence and burden of binge-eating disorder and OSFED globally and present a case for their inclusion in GBD.

**Methods:**

We sourced studies from the GBD 2019 anorexia nervosa and bulimia nervosa epidemiological databases, two systematic reviews that included studies with epidemiological estimates of binge-eating disorder and OSFED, and experts in the field. Studies, published between Jan 1, 1998, and March 1, 2019, were included if they reported non-zero prevalence of two or more eating disorders (anorexia nervosa, bulimia nervosa, binge-eating disorder, or OSFED) and diagnosed cases according to DSM-IV or DSM-5. The proportions of total eating disorder cases that met diagnostic criteria for each individual eating disorder were estimated via network meta-regression and simulation using studies reporting eating disorder prevalence. The global cases unrepresented in GBD 2019 were estimated using the proportions from the simulation and the GBD 2019 eating disorder prevalence. Disability weights for binge-eating disorder and OSFED were then estimated along with disability-adjusted life-years (DALYs). Estimates are presented with 95% uncertainty intervals (UIs).

**Findings:**

54 studies, of which 36 were from high-income countries, were included in the analysis. The number of global eating disorder cases in 2019 that were unrepresented in GBD 2019 was 41·9 million (95% UI 27·9–59·0), and consisted of 17·3 million (11·3–24·9) people with binge-eating disorder and 24·6 million (14·7–39·7) people with OSFED (*vs* 13·6 million [10·2–17·5] people with eating disorders in GBD 2019). Together, binge-eating disorder and OSFED caused 3·7 million (95% UI 2·0–6·5) DALYs globally, bringing the total eating disorder DALYs to 6·6 million (3·8–10·6) in 2019.

**Interpretation:**

Binge-eating disorder and OSFED accounted for the majority of eating disorder cases and DALYs globally. These findings warrant the inclusion of binge-eating disorder and OSFED in future iterations of GBD, which will bring the burden experienced by people living with these disorders to the attention of policy makers with the means to target this burden.

**Funding:**

Queensland Health, Australian National Health and Medical Research Council, and Bill & Melinda Gates Foundation.

## Introduction

Eating disorders manifest as persistent disordered eating behaviours that interfere with daily social and psychological functioning.^1^ These disorders are physically, mentally, and socially disabling,^2^ and are associated with the highest rates of cause-specific mortality among mental disorders.^3^, ^4^ Compared with people without eating disorders, those with these disorders have lower employment participation, greater absenteeism and presenteeism, higher health-care and informal care costs, and lost lifetime earnings for those who die prematurely.^5^

Accurate epidemiological and burden estimates are crucial for understanding the effect of eating disorders on population health, and planning for health systems' response in terms of both prevention interventions and improvement of access to optimal treatment.^6^, ^7^, ^8^ The Global Burden of Diseases, Injuries, and Risk Factors Study (GBD) is an epidemiological study that quantifies mortality and disability from diseases, injuries, and risk factors at global, regional, and national levels. GBD uses the disability-adjusted life-year (DALY) as a metric for burden. One DALY equates to one lost year of healthy life due to either mortality or disability. It is calculated by summing the fatal burden measured in years of life lost (YLLs) with the non-fatal burden measured in years lived with disability (YLDs).

The most recent comprehensive review of the global burden of eating disorders was by Erskine and colleagues, reporting estimates for anorexia nervosa and bulimia nervosa from GBD 2013.^9^ In the latest iteration, GBD 2019,^10^ anorexia nervosa and bulimia nervosa were still the only two eating disorders recognised as causes of burden within the GBD framework. This is because, historically, epidemiological studies have predominantly focused on anorexia nervosa and bulimia nervosa, and often omitted other eating disorders such as binge-eating disorder and other specified feeding or eating disorder (OSFED).^11^, ^12^, ^13^ Both binge-eating disorder and OSFED were formally introduced to diagnostic classification schemes only in 2013 and were both previously captured within the DSM-IV diagnosis of eating disorders not otherwise specified (EDNOS).^1^, ^14^ Binge-eating disorder is characterised by recurrent episodes of compulsive overeating that lead to distress, without attempts to compensate for weight gain. OSFED incorporates several distinct syndromes and is characterised by subclinical (eg, lesser frequency or duration) or atypical symptoms of eating disorders without meeting full criteria for any of the other eating disorders. Previous work has suggested that binge-eating disorder and OSFED are the most common eating disorders,^15^, ^16^ and has shown that up to half of individuals receiving treatment for an eating disorder have a diagnosis of binge-eating disorder or OSFED.^17^

**Research in context**

**Evidence before this study**

We searched PubMed for papers on burden of binge-eating disorder and other specified feeding or eating disorder (OSFED) published up to Nov 10, 2020, using the search string (“Eating disorder”[Title/Abstract] OR “Eating Disorders”[Title/Abstract] OR “Binge eating disorder”[Title/Abstract] OR “OSFED”[Title/Abstract] OR “Other Specified Feeding and Eating Disorder”[Title/Abstract]) AND (“Burden”[Title/Abstract] OR “DALY”[Title/Abstract] OR “YLD”[Title/Abstract] OR “YLL”[Title/Abstract] OR “disability adjusted life year”[Title/Abstract] OR “years lived with disability”[Title/Abstract] OR “years of life lost”[Title/Abstract] OR “disability weight”[Title/Abstract]) without any additional restrictions. This search returned 274 results, of which 12 looked potentially relevant. Of these 12 studies, four reported on results from the Global Burden of Diseases, Injuries, and Risk Factors Study (GBD), focusing only on anorexia nervosa and bulimia nervosa. The remaining studies either reported epidemiology or alternative health metrics (eg, quality of life) on a study sample or compiled these data in a review. No studies of similar aim or methodology to our study were found.

**Added value of this study**

To our knowledge, this study is the first attempt to quantify the global prevalence and burden of binge-eating disorder and OSFED following GBD methodology. The results of this study show that most of the prevalence and burden of eating disorders is not captured by GBD because of its focus on anorexia nervosa and bulimia nervosa. Additionally, to our knowledge, this is the first study to estimate the burden of disorders outside GBD that still follows the GBD framework. This framework includes estimating custom disability weights following GBD assumptions and accounting for comorbidity in the estimation of burden. This method could be used by other researchers to test the feasibility and importance of the inclusion of other disorders in GBD.

**Implications of all the available evidence**

Our results show that the formal inclusion of binge-eating disorder and OSFED in GBD is both feasible and important, and in turn will lead to better representation of eating disorder burden globally. The larger prevalence and burden of eating disorders estimated from this work will have implications for government funding towards eating disorder research and health care. Innovation in the prevention and treatment of eating disorders is crucial given suboptimal remission rates from available treatments. Additionally, this work highlights the need for future epidemiological research to investigate the prevalence of eating disorders in large-scale nationally representative surveys.

We sought to estimate the prevalence and burden due to binge-eating disorder and OSFED globally using methods that adhere to the GBD protocols and framework. In doing so, we evaluated the importance and feasibility of their inclusion within future GBD studies. The availability of burden of disease estimates for binge-eating disorder and OSFED would provide further resources for better understanding the effects of eating disorders on population health, and in determining the best strategies for reducing these effects.

## Methods

### Case definitions

This extension of estimates from GBD 2019 was produced as part of the GBD Collaborator Network and in accordance with the GBD 2019 protocol.^18^ This study adhered to the Guidelines for Accurate and Transparent Health Estimates Reporting (appendix pp 1–2).^19^

We followed DSM-5 case definitions for anorexia nervosa, bulimia nervosa, binge-eating disorder, and OSFED.^1^ OSFED included, but was not limited to, purging disorder, subthreshold bulimia nervosa, subthreshold binge-eating disorder, and atypical anorexia nervosa. Four remaining eating disorders were not included: pica, rumination disorder, avoidant/restrictive food intake disorder, and unspecified feeding or eating disorder.^1^ Their estimated prevalences are unknown and were outside the scope of this analysis.^20^

### Study identification and data extraction

We sourced studies from the epidemiological datasets used to inform the prevalence of anorexia nervosa and bulimia nervosa for GBD 2019.^21^ As part of GBD 2019, systematic reviews were done to find epidemiological data, and this process has been described elsewhere.^9^, ^10^ To summarise, four electronic databases (PubMed, Embase, PsycINFO, and Global Health Data Exchange) were searched, along with grey literature sources and studies suggested by experts. Studies that formed the GBD database reported the prevalence, incidence, remission, or excess mortality of anorexia nervosa or bulimia nervosa diagnosed using DSM or ICD criteria, were published in any language between Jan 1, 1980, and March 1, 2019, and were representative of the general population.^10^, ^22^ Prevalence estimates with a recall period of more than 12 months were excluded.^22^ The epidemiological datasets were last updated in 2019 for GBD 2019. We also sourced studies from two published systematic reviews that searched for epidemiological estimates of binge-eating disorder and OSFED,^15^, ^16^ and studies provided by experts in the field (appendix p 3).

For the current study, studies also had to report non-zero prevalence of two or more eating disorders so that a prevalence ratio could be estimated. Studies that used DSM-III case definitions were excluded from the current study because DSM-III recognised only anorexia nervosa and bulimia nervosa. After removing duplicate studies, the full texts of remaining studies were reviewed against the inclusion criteria for this study. Information on location, sex, age range, prevalence, uncertainty, number of cases, sample size, recall period, disorder, and diagnostic criteria was extracted from each eligible study. Where available, the most detailed data by age and sex were extracted.

### Estimating eating disorder prevalence by diagnosis

Our first step in estimating eating disorder prevalence by diagnosis was to estimate the proportion of all eating disorder cases that met criteria for each eating disorder diagnosis by age and sex, with the aim of using these proportions to adjust age-specific and sex-specific GBD 2019 eating disorder prevalence estimates (which included anorexia nervosa and bulimia nervosa only). There was, however, substantial variation in the number of diagnoses of eating disorders reported across studies, with 20 studies reporting prevalence for two eating disorder diagnoses, 27 studies reporting prevalence for three eating disorder diagnoses, and seven studies reporting prevalence for four eating disorder diagnoses. If we were to meta-analyse each eating disorder diagnosis, the pooled proportions would not sum to 100%. Our solution was to estimate the pooled prevalence ratios between the diagnoses within a network meta-regression. We were then able to calculate the proportions from these pooled ratios. We did a network meta-regression using meta-regression—Bayesian, regularised, and trimmed (MR-BRT)^23^ on the prevalence ratios between bulimia nervosa and the remaining eating disorders (anorexia nervosa, binge-eating disorder, and OSFED). We used MR-BRT for its ability to include covariates in a network meta-analysis. Bulimia nervosa was chosen as the reference because it held the most ratios to other eating disorder diagnoses in the dataset. Each study was given a random intercept, and the meta-regression controlled for age (mid age; ie, the mid-point of the age range of the estimate), sex (percentage female), recall (point *vs* 12 month), diagnostic criteria (DSM-5 *vs* DSM-IV), and location (high income *vs* non-high income). Percentage female was centred at 50% female, and mid age was mean centred. Estimates of the DSM-IV case definition EDNOS that were inclusive of binge-eating disorder were tagged as representing both OSFED and binge-eating disorder in the network. This analysis produced pooled ratios between bulimia nervosa prevalence and the prevalence of anorexia nervosa, binge-eating disorder, and OSFED by age and sex. A Markov chain Monte Carlo (MCMC) simulation was then done to convert the pooled ratios into proportions of eating disorders. 1000 samples were pulled from the probability distributions of pooled ratios and used to calculate the proportion of eating disorder cases that met criteria for bulimia nervosa via the following formula (see appendix p 4 for derivation):

$$P_{\text{BN}}=\frac{R_{\text{AN}}\times R_{\text{BED}}\times R_{\text{OSFED}}}{\left(R_{\text{AN}}+1\right)\times \left(R_{\text{BED}}\times R_{\text{OSFED}}\right)+\left(R_{\text{OSFED}}+R_{\text{BED}}\right)R_{\text{AN}}}$$

where *P*_BN_ represents the proportion of eating disorder cases that met criteria for bulimia nervosa, *R* represents the ratio between bulimia nervosa prevalence and the prevalence of other eating disorders, AN represents anorexia nervosa, and BED represents binge-eating disorder. The proportion of eating disorder cases that met criteria for each remaining eating disorder was then estimated using the following formula:

$$P_{\text{diagnosis}}=\frac{P_{\text{BN}}}{R_{\text{diagnosis}}}$$

Within GBD 2019's estimation of burden, data from epidemiological literature reviews were used to estimate the prevalence of anorexia nervosa and bulimia nervosa via DisMod-MR 2.1.^24^ DisMod-MR 2.1 is a Bayesian disease modelling meta-regression tool that was used to pool epidemiological data across studies to generate prevalence estimates of anorexia nervosa and bulimia nervosa by age, sex, year, and location. Prevalence was estimated for all countries, including those without any available data, by drawing on data from surrounding locations.^10^ Prevalence was constrained to ages of 5–49 years. More detail on DisMod-MR 2.1 has been described elsewhere.^10^, ^24^

To estimate the prevalence of total eating disorder cases (including binge-eating disorder and OSFED) globally in 2019 by age and sex, we did an MCMC simulation across 1000 samples. We divided the 2019 age-sex-specific prevalence of eating disorders estimated from GBD 2019 (consisting of only anorexia nervosa and bulimia nervosa) by the proportion of eating disorder cases that met criteria for anorexia nervosa and bulimia nervosa. Once the prevalence of all eating disorders was estimated, it was multiplied by the proportion of eating disorder cases meeting criteria for binge-eating disorder and OSFED by age and sex.

### Estimating disability weights

Disability weights in GBD 2019 represented health loss from a disorder on a scale from 0 (no health loss) to 1 (equivalent to death). As previously undertaken for GBD, disability weights were derived from community surveys of the general population from Bangladesh, Hungary, Indonesia, Italy, Peru, Sweden, Tanzania, the Netherlands, and the USA, as well as an open internet survey available in English, Spanish, and Mandarin.^10^, ^25^, ^26^ Participants were presented pairs of lay descriptions of health states and asked which of the two was the healthier. Lay descriptions were created by experts, had to be 35 words or less, and use non-clinical language (appendix pp 5–6). The estimated disability weight was 0·224 (95% uncertainty interval [UI] 0·150–0·312) for anorexia nervosa and 0·223 (0·149–0·311) for bulimia nervosa.

Disability weights for binge-eating disorder and OSFED were not included in the disability weight surveys. We followed GBD's approach for estimating disability weights for new causes, which is to make use of data available from the existing list of health states included in the disability weight surveys. As part of the comorbidity correction in GBD, the disability weight of someone with multiple health states is assumed to equal one minus the product of one minus each disability weight for each health state. This process assumes that the disability weight of combined lay descriptions of multiple health states can be estimated via this formula. We therefore assumed that the disability weight of a lay description could be apportioned into components in a similar manner. Hundreds of health states with various lay descriptions have disability weights estimated from the disability weight survey. From these health states, we were able to deduce the disability weight of specific symptoms described in the lay descriptions. The process to estimate disability weights for binge-eating disorder and OSFED is described in detail in the appendix (pp 5–9) and summarised here. To estimate the disability weight of binge-eating disorder, we isolated the disability weight of compensatory behaviours (starving and vomiting) from other health states that were included in the original disability weight surveys, and removed this value from the disability weight of bulimia nervosa. For OSFED, we estimated the proportion of OSFED cases that met criteria for four OSFED diagnoses via a network meta-regression on the prevalence ratios between purging disorder and remaining OSFED diagnoses (subthreshold bulimia nervosa, subthreshold binge-eating disorder, and atypical anorexia nervosa). We then did an MCMC simulation to convert the pooled ratios into proportions of OSFED cases.

We assumed the symptom frequency of subthreshold bulimia nervosa and subthreshold binge-eating disorder to be half the frequency of bulimia nervosa and binge-eating disorder, respectively, based on DSM-5 frequency criteria and research criteria used in epidemiological studies.^27^, ^28^ Given that the disability weight represents the proportion of a year of healthy life lost due to a health state, we assumed that halving the frequency of symptoms described in the health states of bulimia nervosa and binge-eating disorder would halve the proportion of a healthy year lost and therefore their disability weights. To estimate the disability weight for atypical anorexia nervosa, we deduced the disability weight of being underweight from other health states estimated from the disability weight surveys and removed this value from the disability weight of anorexia nervosa. The disability weight of purging disorder was estimated as the disability weight for the bulimia nervosa health state without the binge-eating behaviours. These disability weights were weighted by the OSFED proportions to estimate an overall OSFED disability weight. The disability weight for binge-eating disorder was estimated as 0·045 (95% UI 0·020–0·081) and that for OSFED was estimated as 0·127 (0·086–0·178).

### Estimating burden

DALYs for an eating disorder were the sum of its YLLs and YLDs. YLLs for anorexia nervosa and bulimia nervosa were estimated by multiplying the number of deaths caused directly by each disorder by the remaining life expectancy. GBD 2019 followed ICD coding rules for determining the underlying cause of death.^10^ Despite excess mortality among other mental disorders, anorexia nervosa and bulimia nervosa were the only mental disorders in GBD 2019 identified as underlying causes of death. Deaths by cause, age, sex, location, and year were modelled via the Cause of Death Ensemble modelling (CODEm) strategy, which was informed by the GBD 2019 cause of death database. More detail on CODEm has been described elsewhere.^10^ Estimation of deaths attributable to binge-eating disorder and OSFED would also require models run in CODEm informed by the GBD 2019 cause of death database, which were restricted to disorders included in GBD 2019. Therefore, it was not feasible to estimate YLLs for binge-eating disorder and OSFED and so their burden (ie, DALYs) comprised YLDs only.

YLDs for anorexia nervosa, bulimia nervosa, binge-eating disorder, and OSFED were estimated by multiplying the number of cases of each disorder by their respective disability weights. A comorbidity correction was done as part of GBD 2019 to adjust the YLDs for the co-occurrence of disorders recognised in GBD 2019. The co-occurrence of disorders was simulated within a population of 40 000 simulants for every age, sex, location, and year. Each simulant was given the probability of having each cause of burden (eg, disorders or injuries) equal to its prevalence, and then assigned a cumulative disability weight that was a multiplicative function of all the disability weights with which the simulant was flagged (see appendix p 5 for the formula). The disability weight uniquely attributable to each sequela was adjusted accordingly. The YLD rate for each disorder was then calculated, and the total YLD count was estimated as the rate multiplied by the population. This simulation was done 1000 times, with the uncertainty of prevalence and disability weights propagated throughout the calculations. The reported 95% UIs represent the 25th and 975th ranked results. The comorbidity correction allows for YLDs to be additive across the GBD 2019 cause hierarchy, which classifies mutually exclusive causes into four levels. Anorexia nervosa and bulimia nervosa are level 4 causes, which sit under eating disorders (level 3), which sits under mental disorders (level 2), which sits under non-communicable diseases (level 1). This process is described in detail elsewhere.^10^ YLDs for anorexia nervosa and bulimia nervosa were corrected in this process as part of GBD 2019, but binge-eating disorder and OSFED were not included. To correct binge-eating disorder and OSFED YLDs for comorbidity, we first estimated the raw anorexia nervosa and bulimia nervosa YLDs by multiplying their global age-specific and sex-specific prevalences by their disability weights. We then used the raw anorexia nervosa and bulimia nervosa YLDs and the GBD 2019 anorexia nervosa and bulimia nervosa YLDs to back calculate the age-specific and sex-specific comorbidity corrections that were applied as part of GBD 2019. We then applied this comorbidity correction to our age-specific and sex-specific binge-eating disorder and OSFED YLDs (see appendix pp 10–11 for a detailed description of our comorbidity correction). This correction was done using MCMC simulation across 1000 samples of the eating disorder prevalences and disability weights, and the reported 95% UIs represent the 25th and 975th ranked results.

All statistical analyses were done with R (version 3.6.3)^29^ unless otherwise stated.

### Role of the funding source

The funders of this study had no role in study design, data collection, data analysis, data interpretation, or writing of this report.

## Results

**Figure 1:**
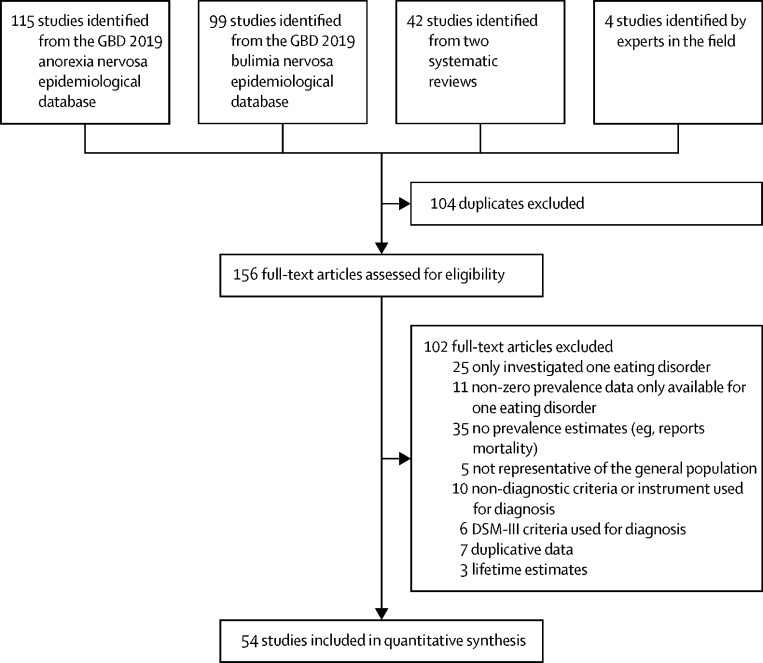
Process of study identification GBD=Global Burden of Diseases, Injuries, and Risk Factors Study.

115 studies were identified from the GBD 2019 anorexia nervosa epidemiological database, 99 from the GBD 2019 bulimia nervosa epidemiological database, 42 from two systematic reviews, and four by experts in the field. Of the 156 studies sourced, 54 met inclusion criteria for the eating disorder diagnosis analysis (figure 1). 36 studies were from high-income countries, seven were from north Africa and the Middle East, and five were from southeast Asia, east Asia, and Oceania. Details of included studies are provided in the appendix (pp 12–21).

The network meta-regression of the prevalence ratios showed that prevalence of anorexia nervosa was lower than the prevalence of bulimia nervosa, and the prevalences of binge-eating disorder and OSFED were higher than that of bulimia nervosa (table). The ratio between anorexia nervosa and bulimia nervosa varied significantly by sex, indicating that the difference in prevalence between the two disorders was smaller among males than among females (β=–0·56 [95% UI −0·97 to −0·14]). The ratio between bulimia nervosa and binge-eating disorder, and between bulimia nervosa and OSFED, varied significantly by mid age, suggesting that the proportion of people with eating disorders who met criteria for binge-eating disorder and OSFED increased with age (β=0·03 [95% UI 0·03 to 0·04] for binge-eating disorder and 0·02 [0·01 to 0·03] for OSFED). The ratios did not vary significantly between high income versus non-high income, and so this covariate was excluded (β=–0·33 [95% UI −0·82 to 0·16] for anorexia nervosa, 0·35 [–0·15 to 0·86] for binge-eating disorder, and −0·36 [–0·96 to 0·25] for OSFED).

**Table:** Estimated prevalence ratios and proportions for eating disorders

		**β (95% UI)**	**Ratio (95% UI)**	**Proportion (95% UI)**
Bulimia nervosa		..	..	0·19 (0·15 to 0·23)
Anorexia nervosa				
	Intercept	−0·75 (−1·03 to −0·47)	2·12 (1·61 to 2·82)	0·09 (0·07 to 0·12)
	Percentage female	−0·56 (−0·97 to −0·14)	..	..
	Mid age	0·01 (−0·00 to 0·02)	..	..
	Past year recall	−0·35 (−0·75 to 0·06)	..	..
	DSM-IV	0·18 (−0·16 to 0·52)	..	..
Binge-eating disorder				
	Intercept	0·47 (0·20 to 0·75)	0·63 (0·47 to 0·82)	0·30 (0·23 to 0·39)
	Percentage female	−0·06 (−0·39 to 0·28)	..	..
	Mid age	0·03 (0·03 to 0·04)	..	..
	Past year recall	0·05 (−0·45 to 0·55)	..	..
	DSM-IV	−0·54 (−0·91 to −0·17)	..	..
Other specified feeding or eating disorder				
	Intercept	0·79 (0·39 to 1·18)	0·46 (0·30 to 0·68)	0·41 (0·31 to 0·52)
	Percentage female	0·33 (−0·10 to 0·75)	..	..
	Mid age	0·02 (0·01 to 0·03)	..	..
	Past year recall	−2·26 (−3·05 to −1·47)	..	..
	DSM-IV	−0·05 (−0·52 to 0·43)	..	..

β values represent the negative log of the prevalence ratio between bulimia nervosa and the remaining eating disorders (anorexia nervosa, binge-eating disorder, and other specified feeding or eating disorder). Ratio and proportion are reported for 50% female population at the mean mid age of 25·5 years (SD 16·2). Percentage female was centred at 50% female, and mid age was mean centred. UI=uncertainty interval.

**Figure 2:**
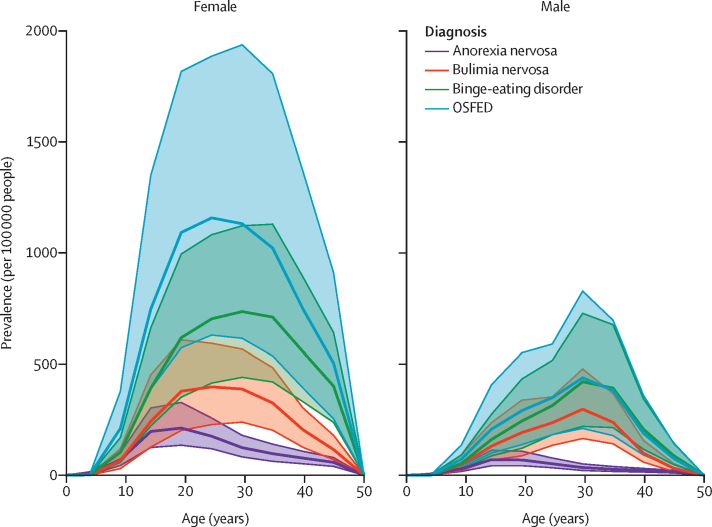
Global prevalence by eating disorder diagnosis, sex, and age in 2019 OSFED=other specified feeding or eating disorder. Shaded areas denote 95% uncertainty intervals.

GBD 2019 estimated that 13·6 million (95% UI 10·2–17·5) people had anorexia nervosa or bulimia nervosa in 2019, equivalent to 176·2 (131·7–225·6) per 100 000 people. We estimated an additional 41·9 million (95% UI 27·9–59·0) prevalent cases of binge-eating disorder and OSFED globally in 2019, equivalent to 541·1 (360·3–762·9) per 100 000 people. Cases consisted of 17·3 million (95% UI 11·3–24·9) people with binge-eating disorder and 24·6 million (14·7–39·7) people with OSFED, equivalent to 223·4 (146·0–322·3) per 100 000 people with binge-eating disorder and 317·8 (190·4–512·5) per 100 000 people with OSFED. The estimated total number of people with eating disorders in 2019 was 55·5 million (95% UI 38·7–75·2), equivalent to 717·3 (500·4–972·1) per 100 000 people. Prevalence by eating disorder, sex, and age is presented in figure 2.

GBD 2019 reported that eating disorders (anorexia nervosa and bulimia nervosa only) were responsible for 2·9 million (95% UI 1·8–4·3) DALYs globally in 2019, equivalent to 37·6 (23·7–56·2) per 100 000 people. We estimated that in 2019 binge-eating disorder accounted for 0·8 million (95% UI 0·3–1·6) DALYs globally (9·8 [3·9–20·2] per 100 000 people), and OSFED for 3·0 million (1·5–5·2) DALYs globally (38·5 [19·7–67·3] per 100 000 people). Altogether, we estimated an additional 3·7 million (95% UI 2·0–6·5) DALYs due to eating disorders (48·3 [25·5–84·3] per 100 000 people) in 2019 compared with DALYs estimated by GBD 2019 for anorexia nervosa and bulimia nervosa.

**Figure 3:**
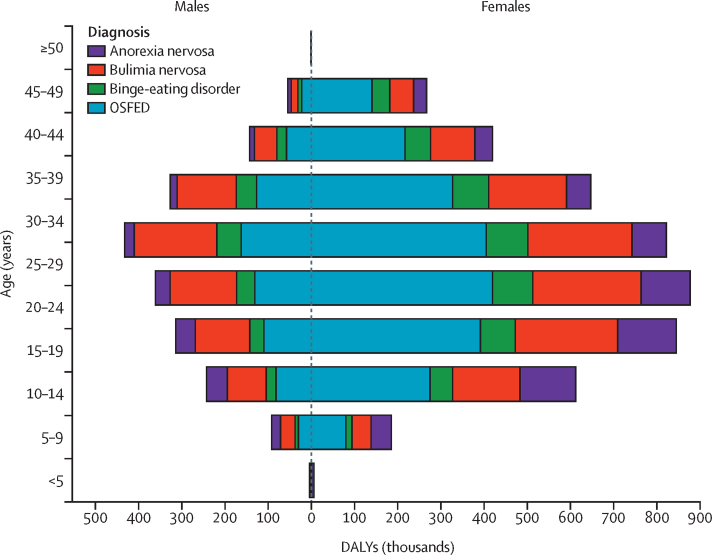
Global DALYs by eating disorder, sex, and age in 2019 DALYs=disability-adjusted life-years. OSFED=other specified feeding or eating disorder.

When combined with GBD 2019 estimates for anorexia nervosa and bulimia nervosa, eating disorders accounted for 6·6 million (95% UI 3·8–10·6) DALYs, equivalent to 85·9 (48·8-137·2) per 100 000 people, in 2019. Burden was higher among females than males (4·7 million [95% UI 2·7–7·6] DALYs *vs* 2·0 million [1·1–3·2] DALYs), and the burden of eating disorders peaked at 25–29 years for females and 30–34 years for males (figure 3).

In GBD 2019, eating disorders (comprising anorexia nervosa and bulimia nervosa only) were ranked the 110th cause of DALYs and 55th cause of YLDs (of 169 level 3 causes) globally in 2019, accounting for 0·1% of all DALYs and 0·3% of YLDs. Inclusion of binge-eating disorder and OSFED would have increased the ranks of eating disorders to 73rd for DALYs and 33rd for YLDs. The more broadly defined eating disorders would have constituted 0·3% of all global DALYs and 0·8% of all global YLDs.

## Discussion

To our knowledge, our study is the first to estimate the prevalence and burden of binge-eating disorder and OSFED globally. By contrast with GBD 2019, the majority of eating disorder cases were classified as binge-eating disorder and OSFED. Their relative contribution to eating disorder prevalence increased with age. This finding showed that, by not including binge-eating disorder and OSFED, a large proportion of the burden due to eating disorders was not captured in GBD 2019. OSFED imposed the most burden owing to its relatively high prevalence and moderate disability weight. Despite having the second highest prevalence of the four eating disorder diagnoses considered, binge-eating disorder carried the least burden, because of its smaller disability weight. Nonetheless, the burden due to binge-eating disorder was not inconsequential, ranking as the 126th of 295 level 4 causes of YLDs globally.

GBD outputs have made important contributions to the public health response to the burden imposed by mental disorders. The inclusion of binge-eating disorder and OSFED as formal GBD causes would provide more representative estimates of the burden imposed by eating disorders, which in turn would have important implications for informing the prioritisation of government funding towards eating disorder research and health care. This research is especially crucial in the context of much-needed innovation in prevention and treatment, given the suboptimal remission rates associated with current recommended evidence-based treatments for eating disorders.^7^, ^30^

Furthermore, our study might provide an impetus for research into binge-eating disorder and OSFED, which have received less attention than other eating disorders. The current study drew on the GBD epidemiological dataset and modelled prevalence and burden for eating disorders. Although GBD is the most comprehensive epidemiological study globally, it is still limited by data coverage. The epidemiological datasets for anorexia nervosa and bulimia nervosa are among the smallest for mental disorders. Diagnostic interviews for eating disorders are often omitted from large nationally representative surveys. Instead, they are often replaced with questionnaires screening for disordered eating behaviours or attitudes.^9^, ^31^, ^32^ The scarcity of diagnostic data for eating disorders is likely to be the result of, and in turn contributes to, the paucity of eating disorder research relative to other mental disorders.^33^, ^34^ One result has been the perceived low prevalence of eating disorders relative to more common mental disorders. The GBD 2019 global prevalence of eating disorders (anorexia nervosa and bulimia nervosa only) was estimated to be less than 0·2% of the population.^10^ The inclusion of binge-eating disorder and OSFED increases the prevalence of eating disorders to 0·7% in our study, in line with drug use disorders, and higher than bipolar disorder, autism spectrum disorder, and conduct disorder.^10^ Inclusion of binge-eating disorder and OSFED in the prevalence of eating disorders in projects such as GBD might promote the incorporation of eating disorders in future nationally representative surveys and in turn improve data coverage and precision of epidemiological and burden estimates.

The current study addressed an important barrier for the inclusion of new disorders in GBD, which is the requirement of appropriate disability weights to estimate YLDs. Disability weights for GBD were estimated via two rounds of surveys in which respondents judged the health status of lay descriptions of health states, across which 235 unique disability weights were estimated.^25^, ^26^ Selecting a health state that aligns with a new disorder can be challenging. A novel approach used in this analysis was to estimate the contribution of each symptom described in the lay descriptions and adjust the disability weights accordingly. This approach does not replace the need for future disability weight surveys to include health states for binge-eating disorder and OSFED against which our estimated disability weights can be validated. However, given that disability weight surveys are not routinely undertaken, this approach enabled us to expand on the GBD cause list in the absence of new surveys.

There are several limitations to this study that affect the precision of estimates. First, the data used to estimate the prevalence of binge-eating disorder and OSFEDs were sourced from the GBD anorexia nervosa and bulimia nervosa epidemiological databases, two published systematic reviews, and expert collaborators. The dataset compiled for this study was sufficient to create and test the method to estimate YLDs for binge-eating disorder and OSFED; however, a new systematic review of binge-eating disorder and OSFED epidemiology is needed. Second, as in GBD 2019, the work presented here is subject to the limitations of the disability weights estimated as part of the disability weights surveys. Disability weights are extremely dependent on the lay descriptions shown to respondents of the disability weights surveys.^25^, ^26^ The brief lay descriptions required focus on the most salient and typical characteristics of a health state, which meant that lay descriptions often omitted detailed symptoms that coincide in complex disorders. Third, the current analysis was done at the global level, with the assumption that the ratios between disorder prevalence are similar across the globe; therefore, the geographical variation of binge-eating disorder and OSFED mirrored that of anorexia nervosa and bulimia nervosa. Previous work has suggested this ratio might vary by region.^35^ We tested the effect of geography on the prevalence ratios, but it was not significant. However, this finding could be limited by the data available, with 36 (67%) of the 54 studies informing the model coming from high-income countries. This limitation could be overcome by modelling the prevalence of binge-eating disorder and OSFED using DisMod-MR 2.1, which is currently limited to causes of burden modelled in GBD. Modelling geographical variation is a strength of DisMod-MR 2.1, and we found sufficient epidemiological data of binge-eating disorder and OSFED to model their prevalence using this software should they be included in future iterations of GBD. Fourth, in our study, DALYs for binge-eating disorder and OSFED consisted of YLDs only. Incorporation of YLLs for binge-eating disorder and OSFED would require models run in CODEm informed by the GBD cause of death database, which is also currently limited to causes of burden modelled in GBD. YLLs due to binge-eating disorder and OSFED could be explored further should they be included in future iterations of GBD. Last, the restriction of eating disorders to the ages of 5–49 years in GBD 2019 might have underestimated the prevalence of eating disorders given evidence that these disorders are prevalent among people older than 49 years.^36^ Inclusion of older age groups would have affected binge-eating disorder and OSFEDs to a greater extent as our model showed that their relative contribution to eating disorder prevalence increases with age. Again, this limitation could be addressed following the formal inclusion of binge-eating disorder and OSFED as causes of burden in GBD. The age restriction for anorexia nervosa and bulimia nervosa should be revisited in future iterations of GBD.

Despite the above limitations, we used available data and novel methodology to estimate the prevalence, disability weights, and burden of binge-eating disorder and OSFED. We aimed to address a key principle of GBD, which states that an uncertain estimate is preferable to no estimate because no estimate is often taken to mean no health loss from that condition.^18^ Indeed, in the absence of these estimates, there is the risk that policy makers and service planners reliant on data from GBD might interpret the absence of binge-eating disorder and OSFED in GBD as no health loss from these disorders. The inclusion of binge-eating disorder and OSFED in GBD will bring recognition of the burden experienced by people living with these disorders to those with the means to target this burden.

## Data sharing

The dataset used to inform this study is available on request to the corresponding author.
