# Supplementary material for: The hidden burden of eating disorders: an extension of estimates from the Global Burden of Disease Study 2019
Source: Lancet Psychiatry. 2021 Apr;8(4):320–8. doi: 10.1016/S2215-0366(21)00040-7 (PMC7973414; doi:10.1016/S2215-0366(21)00040-7)
Supplement: Supplementary appendix [file mmc1.pdf]

THE LANCET
Psychiatry

## Supplementary appendix

This appendix formed part of the original submission and has been peer reviewed. We post it as supplied by the authors.

Supplement to: Santomauro DF, Melen S, Mitchison D, Vos T, Whiteford H, Ferrari AJ. The hidden burden of eating disorders: an extension of estimates from the Global Burden of Disease Study 2021. *Lancet Psychiatry* 2021; published online March 3. https://doi.org/10.1016/S2215-0366(21)00040-7.

## eTable 1: Guidelines for Accurate and Transparent Health Estimates Reporting (GATHER) checklist

| Item # | Checklist item | Location |
|---|---|---|
| **Objectives and funding** | | |
| 1 | Define the indicator(s), populations (including age, sex, and geographic entities), and time period(s) for which estimates were made. | Introduction pp 1-2<br>Methods: *Case definition* p 2<br>Methods: *Estimating eating disorder prevalence by diagnosis* pp 3-4<br>Methods: *Estimating Burden* p 4-5 |
| 2 | List the funding sources for the work. | Acknowledgements p 8 |
| **Data Inputs** | | |
| ***For all data inputs from multiple sources that are synthesized as part of the study:*** | | |
| 3 | Describe how the data were identified and how the data were accessed. | Methods: *Study identification and data extraction* p 2-3 |
| 4 | Specify the inclusion and exclusion criteria. Identify all ad-hoc exclusions. | Methods: *Case definitions* p 2<br>Methods: *Study identification and data extraction* p 2-3 |
| 5 | Provide information on all included data sources and their main characteristics. For each data source used, report reference information or contact name/institution, population represented, data collection method, year(s) of data collection, sex and age range, diagnostic criteria or measurement method, and sample size, as relevant. | eTable 8 appendix pp 12-21 |
| 6 | Identify and describe any categories of input data that have potentially important biases (e.g., based on characteristics listed in item 5). | Methods: *Case definitions* p 2<br>Methods: *Estimating eating disorder prevalence by diagnosis* pp 3-4 |
| ***For data inputs that contribute to the analysis but were not synthesized as part of the study:*** | | |
| 7 | Describe and give sources for any other data inputs. | Supplementary Methods: *Estimation of the disability weights for binge-eating disorders and other specified feeding and eating disorders* appendix pp 5-9 |
| ***For all data inputs:*** | | |
| 8 | Provide all data inputs in a file format from which data can be efficiently extracted (e.g., a spreadsheet rather than a PDF), including all relevant meta-data listed in item 5. For any data inputs that cannot be shared because of ethical or legal reasons, such as third-party ownership, provide a contact name or the name of the institution that retains the right to the data. | *GBD 2019 epidemiological datasets are available for download here:*<br>*http://ghdx.healthdata.org/gbd-2019/data-input-sources*<br>*Disability weights are available for download here:*<br>*http://ghdx.healthdata.org/record/ihme-data/gbd-2019-disability-weights*<br>*Data prepped for analysis is available as a csv on request to corresponding author* |

| | | |
|---|---|---|
| **Data analysis** | | |
| 9 | Provide a conceptual overview of the data analysis method. A diagram may be helpful. | Methods: *Estimating eating disorder prevalence by diagnosis* pp 3-4 |
| 10 | Provide a detailed description of all steps of the analysis, including mathematical formulae. This description should cover, as relevant, data cleaning, data pre-processing, data adjustments and weighting of data sources, and mathematical or statistical model(s). | Methods: *Estimating eating disorder prevalence by diagnosis* pp 3-4<br>Methods: *Estimating disability weights* p 4<br>Methods: *Estimating burden* p 4-5<br>Supplementary Methods: *Derivation of formula for estimating the proportion of eating disorder cases that met criteria for bulimia nervosa from the prevalence ratios between bulimia nervosa and the other eating disorders* appendix p 4<br>Supplementary Methods: *Estimation of the disability weights for binge-eating disorders and other specified feeding and eating disorders* appendix pp 5-9<br>Supplementary Methods: *Comorbidity correction for binge-eating disorder and other specified feeding and eating disorders* appendix pp 10-11 |
| 11 | Describe how candidate models were evaluated and how the final model(s) were selected. | Methods: *Estimating eating disorder prevalence by diagnosis* pp 3-4 |
| 12 | Provide the results of an evaluation of model performance, if done, as well as the results of any relevant sensitivity analysis. | N/A |
| 13 | Describe methods for calculating uncertainty of the estimates. State which sources of uncertainty were, and were not, accounted for in the uncertainty analysis. | Methods: *Estimating eating disorder prevalence by diagnosis* pp 3-4<br>Methods: *Estimating disability weights* p 4<br>Methods: *Estimating burden* p 4-5 |
| 14 | State how analytic or statistical source code used to generate estimates can be accessed. | *R scripts available on request to corresponding author* |
| **Results and Discussion** | | |
| 15 | Provide published estimates in a file format from which data can be efficiently extracted. | *Estimates produced at the global level and can be efficiently extracted from text.* |
| 16 | Report a quantitative measure of the uncertainty of the estimates (e.g. uncertainty intervals). | *Prevalence, disability weights, YLDs, and DALYs are reported with 95% uncertainty intervals.* |
| 17 | Interpret results in light of existing evidence. If updating a previous set of estimates, describe the reasons for changes in estimates. | Discussion pp 6-8 |
| 18 | Discuss limitations of the estimates. Include a discussion of any modelling assumptions or data limitations that affect interpretation of the estimates. | Discussion pp 6-8 |

## Supplementary Methods: Study identification and inclusion criteria and data extraction for studies sourced outside the GBD epidemiological database.

Data sources for this project were obtained from the epidemiological datasets used to inform the prevalence of anorexia nervosa and bulimia nervosa for GBD 2019, from two published systematic reviews, and from experts in the field. Detail on the epidemiological datasets used to inform the prevalence of anorexia nervosa and bulimia nervosa for GBD 2019 is described in the main text of the manuscript (page 3). The two published systematic reviews were Erskine and colleagues[1] and Lindvall Dahlgren and colleagues.[2]

Erskine and colleagues[1] searched PubMed, Embase, and PsycINFO for studies reporting prevalence, incidence, remission, and excess mortality of binge-eating disorder published between January 1980 (day not reported) and December 2017 (day not reported). Samples had to be representative of the general population and cases had to be diagnosed using DSM-IV-TR research criteria or DSM-5 criteria. Estimates of lifetime prevalence, intervention studies, and randomised controlled trials were excluded.

Lindvall Dahlgren and colleagues[2] searched PubMed for studies reporting prevalence of eating disorders among non-clinical samples published in a peer reviewed journal between 2012 (day and month not reported) and February 2017 (day not reported). Studies had to be published in English or have a published English translation. Cases had to be diagnosed using DSM-5 criteria. Studies reporting prevalence on clinical samples were excluded. Editorials, commentaries, and reviews (including systematic reviews) were also excluded.

Sources were obtained from experts in the field via two pathways. First, the GBD team engages with an extensive network of collaborators from various parts of the world, on a regular basis. Those with expertise in mental health, or eating disorders more specifically, were given the opportunity to review epidemiological datasets and provide additional data. Second, a subset of these collaborators who actively contribute to this topic area and have ongoing links with the research team were approached for additional data relevant to this analysis. Communication with experts in the field was ongoing for the duration of the project. All sources provided were evaluated against the previously mentioned inclusion criteria.

## Supplementary Methods: Derivation of formula for estimating the proportion of eating disorder cases that met criteria for bulimia nervosa from the prevalence ratios between bulimia nervosa and the other eating disorders.

**Step 1: Assumed the proportions of the eating disorder diagnoses sum to 1**

$$P_{BN} + P_{AN} + P_{BED} + P_{OSFED} = 1$$

Where *P* represents the proportion of each individual eating disorder, *BN* is bulimia nervosa, *AN* is anorexia nervosa, *BED* is binge-eating disorder, and *OSFED* is other specified feeding and eating disorders (OSFED).

**Step 2: Substituted the proportion of the diagnoses with prevalence ratios to bulimia nervosa**

$$R_i = \frac{P_{BN}}{P_i}$$

$$P_{BN} + \frac{P_{BN}}{R_{AN}} + \frac{P_{BN}}{R_{BED}} + \frac{P_{BN}}{R_{OSFED}} = 1$$

Where *R* represents the prevalence ratio between diagnosis *i* and bulimia nervosa.

**Step 3: Created common denominators to produce a single fraction**

$$\frac{P_{BN} \times R_{AN}}{R_{AN}} + \frac{P_{BN}}{R_{AN}} + \frac{P_{BN} \times R_{OSFED}}{R_{BED} \times R_{OSFED}} + \frac{P_{BN} \times R_{BED}}{R_{OSFED} \times R_{BED}} = 1$$

$$\frac{P_{BN} \times R_{AN} + P_{BN}}{R_{AN}} + \frac{P_{BN} \times R_{OSFED} + P_{BN} \times R_{BED}}{R_{BED} \times R_{OSFED}} = 1$$

$$\frac{(P_{BN} \times R_{AN} + P_{BN}) \times (R_{BED} \times R_{OSFED}) + (P_{BN} \times R_{OSFED} + P_{BN} \times R_{BED}) \times R_{AN}}{R_{AN} \times R_{BED} \times R_{OSFED}} = 1$$

**Step 4: Made the fraction a multiplicative of $P_{BN}$**

$$P_{BN} \times \left(\frac{(R_{AN} + 1) \times (R_{BED} \times R_{OSFED}) + (R_{OSFED} + R_{BED}) \times R_{AN}}{R_{AN} \times R_{BED} \times R_{OSFED}}\right) = 1$$

**Step 5: Solved for $P_{BN}$**

$$P_{BN} = \frac{1}{\left(\frac{(R_{AN} + 1) \times (R_{BED} \times R_{OSFED}) + (R_{OSFED} + R_{BED}) \times R_{AN}}{R_{AN} \times R_{BED} \times R_{OSFED}}\right)}$$

$$P_{BN} = \frac{R_{AN} \times R_{BED} \times R_{OSFED}}{(R_{AN} + 1) \times (R_{BED} \times R_{OSFED}) + (R_{OSFED} + R_{BED}) \times R_{AN}}$$

## Supplementary Methods: Estimation of the disability weights for binge-eating disorders and other specified feeding and eating disorders

Disability weights represent health loss due to a disorder on a scale of 0 (no health loss) and 1 (equivalent to death). Disability weights for the Global Burden of Disease Study (GBD) were derived from community surveys of the general population across 9 countries (Bangladesh, Hungary, Indonesia, Italy, Peru, Sweden, Tanzania, the Netherlands, and the USA) and an open internet survey available in English, Spanish, and Mandarin.[3,4] Participants of these surveys were presented pairs of lay descriptions of health states and asked which of the two health states was the healthier. The lay descriptions for each health state were created by experts, had to be 35 words or less, and use non-clinical language. The name of the health state was not included in the lay description to reduce bias. Responses were anchored between 0 and 1 using additional population health equivalence questions that compared the benefits of lifesaving and disease-prevention programs for health states.

As part of the comorbidity correction in GBD, the cumulative (i.e., total) disability experienced by someone living with multiple disorders is equal to one minus the product of one minus each disability weight for each disorder:

$$DW_{cumulative} = 1 - \prod (1 - DW_i)$$

Where *DW* represents disability weight and *i* represents each disorder contributing to the cumulative disability weight. This method assumes the cumulative disability weight of combined health states, and their respective lay descriptions, can be estimated via the above formula. For example, someone with speech problems (disability weight = 0·051, lay description = “has difficulty speaking, and others find it difficult to understand”) and stage 4 chronic kidney disease (disability weight = 0·104, lay description = “tires easily, has nausea, reduced appetite and difficulty sleeping”) would have a cumulative disability weight of 0·150 and the lay description of “tires easily, has nausea, reduced appetite, difficulty sleeping, difficulty speaking, and others find it difficult to understand”. Extending this assumption, we assumed the disability weight for any health state is a cumulative disability weight of the symptoms described in its lay description.

### Estimation of the disability weight for binge-eating disorder

The disability weight for binge-eating disorder was assumed to be the disability weight for bulimia nervosa without starving and vomiting / purging behaviours.

***Step 1: Selected the health state with closest lay description to binge-eating disorder.*** First, the *bulimia nervosa* health state was selected as having the closest lay description to what would describe binge-eating disorder. The lay description was then broken down into individual symptoms (see eTable 2).

**eTable 2: Lay description and disability weight for bulimia nervosa health state**

| Health state | Lay description | Disability weight (95% UIs) | Symptoms |
|---|---|---|---|
| Bulimia nervosa | has uncontrolled overeating followed by guilt, starving, and vomiting to lose weight. | 0·223 (0·149-0·311) | Uncontrolled overeating |
| | | | Guilt |
| | | | Starving |
| | | | Vomiting |

UI = Uncertainty interval

***Step 2: Estimated the disability weight for the symptom “starving”.*** Another health state with *starving* in its lay description was selected in order to isolate the disability weight for this symptom. *Anorexia nervosa* was selected and again its lay description was broken down into individual symptoms. Symptoms *very thin* and *weak* were assigned the disability weight for *Severe wasting* as they were conceptually similar to the lay description “*is extremely skinny and has no energy”* (see eTable 3). *Anxious* was assigned the disability weight *Anxiety disorders, mild*. *Anxiety disorders, mild* was selected over the *Anxiety disorders, moderate* or *Anxiety disorders, severe* because the latter two had very large disability weights and resulted in a negative disability weight for *starving*.

**eTable 3: Lay description and disability weight for anorexia nervosa health state, its symptoms, and their assigned health states**

| Health state | Lay description | Disability weight (95% UIs) | Symptoms | Symptom health state | Symptom lay description | Symptom disability weight (95% UIs) |
|---|---|---|---|---|---|---|
| Anorexia nervosa | feels an overwhelming need to starve and exercises excessively to lose weight. The person is very thin, weak and anxious. | 0·224 (0·15-0·312) | Overwhelming need to starve and exercises excessively to lose weight | | | |
| | | | Very thin | Severe wasting | is extremely skinny and has no energy. | 0·128 (0·082-0·183) |
| | | | Weak | | | |
| | | | Anxious | Anxiety disorders, mild | feels mildly anxious and worried, which makes it slightly difficult to concentrate, remember things, and sleep. The person tires easily but is able to perform daily activities. | 0·030 (0·018-0·046) |

UI = Uncertainty interval

The disability weight for *overwhelming need to starve and exercises excessively to lose weight* was back-calculated using the cumulative disability weight for *Anorexia nervosa* and the disability weights for its symptoms *very thin and* weak and *anxious*:

$$DW_{Starving} = 1 - \frac{(1 - DW_{Anorexia\ nervosa})}{(1 - DW_{Very\ thin\ and\ weak}) \times (1 - DW_{Anxious})}$$

The disability weight for *starving* was 0·085 (95% uncertainty interval [UI]: 0·058-0·119).

***Step 3: Estimate the disability weight for the symptom "vomiting".*** Another health state with *vomiting* in its lay description was selected in order to isolate the disability weight for this symptom. *Poisoning (short term with or without treatment)* was selected and again its lay description was broken down into individual symptoms. The symptom *drowsiness* was assigned the disability weight for *Sleep apnoea* as the lay description for that health state describes a state of drowsiness (see eTable 4). The symptom *stomach pain* was assigned the disability weight for *Intestinal nematode infections, symptomatic* for its detailed description of stomach pain.

**eTable 4: Lay description and disability weight for Poisoning (short term with or without treatment) health state, its symptoms, and their assigned health states**

| Health state | Lay description | Disability weight (95% UI) | Symptoms | Symptom health state | Symptom lay description | Symptom disability weight (95% UI) |
|---|---|---|---|---|---|---|
| Poisoning (short term with or without treatment) | has drowsiness, stomach pain and vomiting. | 0·163 (0·109-0·227) | Drowsiness | Sleep apnoea | feels sleepy during the day and has difficulty concentrating. | 0·032 (0·018-0·050) |
| | | | Stomach Pain | Intestinal nematode infections, symptomatic | has cramping pain and a bloated feeling in the belly. | 0·027 (0·015-0·043) |
| | | | Vomiting | | | |

UI = Uncertainty interval

The disability weight for *vomiting* was then back-calculated using the cumulative disability weight for *Poisoning (short term with or without treatment)* and the disability weights for its symptoms *drowsiness* and *stomach pain*:

$$DW_{vomiting} = 1 - \frac{(1 - DW_{Poisoning\ (short\ term\ with\ or\ without\ treatment)})}{(1 - DW_{Drowsiness}) \times (1 - DW_{Stomach\ pain})}$$

The disability weight for *vomiting* was estimated to be 0·112 (95% UI: 0·079-0·149).

***Step 4: Remove the disability of "vomiting" and "starving" from the "bulimia nervosa" disability weight.*** The disability weight for binge-eating disorder was back-calculated using the cumulative disability weight for *bulimia nervosa* and the disability weight for its symptoms *starving* and *vomiting*.

$$DW_{Binge-eating\ disorder} = 1 - \frac{(1 - DW_{Bulimia\ nervosa})}{(1 - DW_{Starving}) \times (1 - DW_{Vomiting})}$$

Final disability weight for binge-eating disorder was 0·045 (95% UI: 0·020-0·081).

## Estimation of disability weight for other specified feeding and eating disorders (OSFED)

The estimation of a disability weight for OSFED was challenging as it is an umbrella for several heterogeneous eating disorders. Three studies sourced from the GBD 2019 epidemiological dataset,[5-7] and four studies provided by expert collaborators[8-11] suggest the four primary OSFED disorders were subthreshold bulimia nervosa, subthreshold binge-eating disorder, purging disorder, and atypical anorexia nervosa. All seven studies used DSM-5 criteria. There were three steps to the estimation of an overall disability weight for OSFED. First, disability weights for the four primary OSFED diagnoses were estimated. Second, the proportion of OSFED cases meeting criteria for each OSFED diagnosis was estimated in a network meta-analysis of prevalence ratios sourced from the above seven studies. Finally, the OSFED diagnosis disability weights were then weighted according to their estimated proportions of total OSFED cases.

### Disability weights for subthreshold bulimia nervosa and subthreshold binge-eating disorder

The main difference in DSM-5 criteria between bulimia nervosa vs subthreshold bulimia nervosa, and binge-eating disorder vs subthreshold binge-eating disorder, is the frequency and / or duration of the primary symptoms.[12] An individual must display at least one uncontrollable binge-eating episode and one compensatory behaviour per week for at least three months to meet criteria for bulimia nervosa. If an individual displays a smaller frequency of these episodes and behaviours, or experienced them for less than three months, then they would receive a diagnosis of subthreshold bulimia nervosa instead (assuming they did not meet full criteria for another eating disorder). Similarly, an individual must display at least one uncontrollable binge-eating episode per week (without compensatory behaviours) for at least three months to meet criteria for binge-eating disorder. If an individual displays a smaller frequency of these episodes or experienced them for less than three months, then they would receive a diagnosis of subthreshold binge-eating disorder.

There is no explicit minimum frequency of symptoms of the subthreshold disorders in the DSM-5. However, several epidemiological studies have operationalised DSM-5 criteria as at least 2 episodes / behaviours in the past month.[9,11] This aligns with the original DSM-IV-TR criteria for the subthreshold disorder,[13] and is also the mid-point between the minimum (once per month) and maximum (three times per month) possible frequency of episodes / behaviours to meet DSM-5 criteria for the subthreshold disorder. We therefore made the crude assumption that on average someone that meets criteria for the subthreshold disorders likely experiences symptoms at roughly half the frequency as someone that meets full criteria for bulimia nervosa or binge-eating disorder. Given the disability weight equates to the proportion of a year of healthy life lost due to the disorder, we can make a crude assumption that halving the frequency of symptoms halves the proportion of a healthy year lost due to the disorder. In turn, we assumed the disability weight of the subthreshold disorders were equal to half the disability weight for the full disorders:

$$DW_{Subthreshold\ bulimia\ nervosa} = \frac{DW_{bulimia\ nervosa}}{2}$$

$$DW_{Subthreshold\ binge-eating\ disorder} = \frac{DW_{binge-eating\ disorder}}{2}$$

### Disability weight for atypical anorexia nervosa

The disability weight for atypical anorexia nevosa was assumed to be the disability weight for anorexia nervosa without the description “The person is very thin” in the lay description.

***Step 1: Selected the health state with closest lay description to atypical anorexia.*** First, the *anorexia nervosa* health state was selected as having the closest lay description to what would describe atypical anorexia nervosa. The lay description was the broken down into individual symptoms (see eTable 5).

**eTable 5: Lay description and disability weight for anorexia nervosa health state**

| Health state | Lay description | Disability weight (95% UIs) | Symptoms |
|---|---|---|---|
| Anorexia nervosa | feels an overwhelming need to starve and exercises excessively to lose weight. The person is very thin, weak and anxious. | 0·224 (0·15-0·312) | Overwhelming need to starve and exercises excessively to lose weight |
| | | | Very thin |
| | | | Weak |
| | | | Anxious |

UI = Uncertainty interval

***Step 2: Estimated the disability weight for the symptom "very thin".*** The health state *Severe wasting* was selected as the lay description "*is extremely skinny and has no energy*" was conceptually similar to the symptoms *very thin and weak.* Its lay description was broken down into individual symptoms and *no energy* was assigned the disability weight for *Anemia, moderate* because its lay description was a detailed account of someone lacking energy (see eTable 6).

**eTable 6: Lay description and disability weight for Severe wasting health state, its symptoms, and their assigned health states**

| Health state | Lay description | Disability weight (95% UIs) | Symptoms | Symptom health state | Symptom lay description | Symptom disability weight (95% UIs) |
|---|---|---|---|---|---|---|
| Severe wasting | is extremely skinny and has no energy. | 0·128 (0·082-0·183 | Extremely skinny | | | |
| | | | No energy | Anemia, moderate | feels moderate fatigue, weakness, and shortness of breath after exercise, making daily activities more difficult | 0·052 (0·034-0·076) |

UI = Uncertainty interval

The disability weight for *very thin* was then back-calculated using the cumulative disability weight for *Severe wasting* and the disability weight for its symptom *no energy*:

$$DW_{Very\ thin} = 1 - \frac{\left(1 - DW_{Severe\ wasting}\right)}{\left(1 - DW_{No\ energy}\right)}$$

The disability weight for *very thin* was 0·080 (95% UI: 0·044-0·129).

***Step 3: Removed the disability of "very thin" from the "anorexia nervosa" disability weight.*** The disability weight for atypical anorexia nervosa was back-calculated using the cumulative disability weight for *anorexia nervosa* and the disability weight for its symptom *very thin*:

$$DW_{Atypical\ anorexia\ nervosa} = 1 - \frac{(1 - DW_{Anorexia\ nervosa})}{\left(1 - DW_{Very\ thin}\right)}$$

Final disability weight for atypical anorexia nervosa was 0·157 (95% UI: 0·098-0·231).

**Disability weight for purging disorder**

This process assumes that the primary difference in the lay description between bulimia nervosa and purging disorder would be the removal of the description "has uncontrolled overeating followed by guilt". The remaining lay description is equivalent to the lay description that is removed from *bulimia nervosa* to estimate the disability weight for binge-eating disorder. Therefore the disability weight for purging disorder was the disability weight of *bulimia nervosa* with the disability of binge-eating disorder removed.

$$DW_{Purging\ disorder} = 1 - \frac{(1 - DW_{Bulimia\ nervosa})}{\left(1 - DW_{binge-eating\ disorder}\right)}$$

Final disability weight for purging disorder was 0·195 (95% UI: 0·135-0·266).

**Pooled disability weight for OSFED**

A network meta-analysis was conducted via Meta Regression: Bayesian, Regularized, Trimmed[14] (MR-BRT) on the prevalence ratios between purging disorder and the remaining OSFED disorders sourced from the seven studies that reported OSFED diagnosis prevalence. Purging disorder was chosen as the reference in the analysis as it contained the most comparisons in the dataset. Ratios were nested within studies. This analysis produced pooled ratios between purging disorder prevalence and the prevalence of subthreshold bulimia nervosa, subthreshold binge-eating disorder, and atypical anorexia nervosa. A Markov Chain Monte Carlo (MCMC) simulation was then conducted to convert the pooled ratios into proportions of OSFED cases. One thousand samples were pulled from the probability distributions of pooled ratios. The predicted ratios were then used to estimate the proportion of OSFED cases that met criteria for each diagnosis. First, the proportion of OSFED cases that met criteria for purging disorder was estimated using the same formula described in Appendix 1 (but substituting bulimia nervosa with purging disorder, and the other eating disorder diagnoses with the remaining OSFED diagnoses). Then the proportion of OSFED cases that met criteria for the remaining OSFED diagnoses were estimated by dividing the proportion of OSFED cases that met criteria for purging disorder by the respective ratios predicted from the network meta-analysis. A weighted disability weight was then calculated using the following formula:

$$DW_{OSFED} = \sum P_i \times DW_i$$

Where $P$ represents the proportion of OSFED cases that met criteria for OSFED disorder $i$, and $DW_{OSFED}$ represents the disability weight for OSFED disorder $i$. The pooled disability weight for OSFED is reported in the eTable 7.

**eTable 7: The OSFED disability weights and the proportion of OSFED cases meeting criteria for each diagnosis**

| **OSFED disorder** | **Proportion of OSFED cases (95% UI)** | **Disability weight (95% UI)** | **Pooled disability weight (95% UI)** |
|---|---|---|---|
| Subthreshold bulimia nervosa | 0·163 (0·121-0·214) | 0·112 (0·075-0·155) | 0·127 (0·086-0·178) |
| Subthreshold binge-eating disorder | 0·224 (0·188-0·264) | 0·023 (0·010-0·040) | |
| Atypical anorexia nervosa | 0·353 (0·298-0·410) | 0·157 (0·098-0·231) | |
| Purging disorder | 0·260 (0·233-0·288) | 0·187 (0·131-0·250) | |

OSFED = Other specified feeding and eating disorder, UI = Uncertainty interval.

## Supplementary Methods: Comorbidity correction for binge-eating disorder and other specified feeding and eating disorders

To adjust for comorbidity in GBD, a micro-simulation called "COMO" is conducted they 40,000 individuals are simulated for every age, sex, location, and year. Each simulant is given the probability of acquiring each cause of burden in GBD equal to the prevalence of each cause of burden. A cumulative disability weight is then estimated for each simulant using the following formula:

$$DW_{cumulative} = 1 - \prod_{k=i}^{j}(1 - DW_k)$$

Where $DW_{\text{cumulative}}$ is the cumulative disability weight for the simulant, and $DW_k$ is the disability weight for the $k^{th}$ cause of burden that the simulant has acquired. The final disability weight for each cause of burden is then calculated using the following formula:

$$AdjDW_k = DW_{cumulative} \times \frac{DW_k}{\sum_{k=i}^{k=j} DW_k}$$

Where $AdjDW_k$ is the comorbidity adjusted disability weight for the $k^{th}$ cause of burden. Whilst in GBD this is done at the simulant level, these formulas are also valid at the population level. To estimate the comorbidity correction for binge-eating disorder and OSFED in the current study, the above formulas were combined into a single formula:

$$AdjDW_{ED} = (1 - (1 - DW_{ED}) \times (1 - DW_{como})) \times \frac{DW_{ED}}{DW_{ED} + DW_{como}}$$

Where $AdjDW_{ED}$ is the comorbidity adjusted disability weight for the eating disorders, $DW_{ED}$ is the raw disability weight for the eating disorders, and $DW_{como}$ is the cumulative disability weight of the comorbidities. $AdjDW_{ED}$ was estimated by dividing the YLDs caused by eating disorders in GBD 2019 by the sum of the prevalence of anorexia nervosa and bulimia nervosa by age and sex. $DW_{ED}$ was estimated by calculating the summing the product of the disability weights for anorexia nervosa and bulimia nervosa by their respective prevalence estimates to get crude YLDs for eating disorders, and then dividing the the crude YLDs by the sum of the anorexia nervosa and bulimia nervosa prevalence. This formula was then solved to estimate the cumulative disability weight of the comorbidities:

**Step 1: Multiply both sides of the equation with the denominator of the fraction**

$$AdjDW_{ED} \times (DW_{ED} + DW_{como}) = (1 - (1 - DW_{ED}) \times (1 - DW_{como})) \times DW_{ED}$$

**Step 2: Expand brackets**

$$AdjDW_{ED}DW_{ED} + AdjDW_{ED}DW_{como} = (1 - (1 - DW_{como} - DW_{ED} + DW_{ED}DW_{como})) \times DW_{ED}$$

$$AdjDW_{ED}DW_{ED} + AdjDW_{ED}DW_{como} = (1 - 1 + DW_{como} + DW_{ED} - DW_{ED}DW_{como}) \times DW_{ED}$$

$$AdjDW_{ED}DW_{ED} + AdjDW_{ED}DW_{como} = (DW_{como} + DW_{ED} - DW_{ED}DW_{como}) \times DW_{ED}$$

$$AdjDW_{ED}DW_{ED} + AdjDW_{ED}DW_{como} = DW_{ED}DW_{como} + DW_{ED}^2 - DW_{ED}^2DW_{como}$$

**Step 3: Rearrange equation to make a function of $DW_{\text{como}}$**

$$AdjDW_{ED}DW_{ED} - DW_{ED}^2 = DW_{ED}DW_{como} - DW_{ED}^2DW_{como} - AdjDW_{ED}DW_{como}$$

$$AdjDW_{ED}DW_{ED} - DW_{ED}^2 = DW_{como}(DW_{ed} - DW_{ED}^2 - AdjDW_{ED})$$

**Step 4: Solve for $DW_{como}$**

$$\frac{AdjDW_{ED}DW_{ED} - DW_{ED}^{2}}{DW_{ED} - DW_{ED}^{2} - AdjDW_{ED}} = DW_{como}$$

Once the cumulative disability weight of the comorbidities was estimated by age and sex, the comorbidity corrected disability weight for binge-eating disorder and OSFED were each estimated using the standard comorbidity adjustment formula used in GBD:

$$AdjDW_{k} = 1 - (1 - DW_{k}) \times (1 - DW_{como})$$

## eTable 8: Characteristics of studies and data included in the network meta-regression of prevalence ratios

| Study | Case name | Years | Ages | Sex | % female | Prevalence / 100,000 persons | SE | Sample size | Recall | Case definition | Location |
|---|---|---|---|---|---|---|---|---|---|---|---|
| Aalto-Setälä et al. (2001)[15] | Anorexia Nervosa | 1995-1995 | 20-24 | Female | 100·0 | 700·0 | 510·2 | 414 | Point | DSM-IV | Finland |
| | Anorexia Nervosa | 1995-1995 | 20-24 | Female | 100·0 | 700·0 | 510·2 | 414 | Point | DSM-IV | Finland |
| | Bulimia Nervosa | 1995-1995 | 20-24 | Female | 100·0 | 2100·0 | 867·4 | 414 | Point | DSM-IV | Finland |
| | OSFED + BED | 1995-1995 | 20-24 | Female | 100·0 | 2500·0 | 892·9 | 414 | Point | DSM-IV | Finland |
| Ackard et al. (2007)[16] | Anorexia Nervosa | 1998-1999 | 11-18 | Female | 100·0 | 42·1 | 42·1 | 2373 | Point | DSM-IV | Minnesota |
| | Anorexia Nervosa | 1998-1999 | 11-18 | Female | 100·0 | 42·1 | 42·1 | 2373 | Point | DSM-IV | Minnesota |
| | BED | 1998-1999 | 11-18 | Female | 100·0 | 337·1 | 119·0 | 2373 | Point | DSM-IV | Minnesota |
| | BED | 1998-1999 | 11-18 | Male | 0·0 | 1896·3 | 280·0 | 2373 | Point | DSM-IV | Minnesota |
| | Bulimia Nervosa | 1998-1999 | 11-18 | Female | 100·0 | 337·1 | 119·0 | 2373 | Point | DSM-IV | Minnesota |
| | Bulimia Nervosa | 1998-1999 | 11-18 | Male | 0·0 | 168·6 | 84·2 | 2373 | Point | DSM-IV | Minnesota |
| Alatiq et al. (2017)[17] | Anorexia Nervosa | 2015-2016 | 15-99 | Female | 100·0 | 84·3 | 42·1 | 4745 | Point | DSM-IV | Saudi Arabia |
| | Bulimia Nervosa | 2015-2016 | 15-99 | Female | 100·0 | 484·7 | 100·8 | 4745 | Point | DSM-IV | Saudi Arabia |
| Allen et al. (2013)[5] | Anorexia Nervosa | 2004-2004 | 14-14 | Female | 100·0 | 300·0 | 206·3 | 703 | Point | DSM-5 | Australia |
| | Anorexia Nervosa | 2004-2004 | 14-14 | Female | 100·0 | 300·0 | 206·3 | 703 | Point | DSM-5 | Australia |
| | Anorexia Nervosa | 2007-2007 | 17-17 | Female | 100·0 | 1400·0 | 443·1 | 703 | Point | DSM-5 | Australia |
| | Anorexia Nervosa | 2010-2010 | 20-20 | Female | 100·0 | 600·0 | 291·3 | 703 | Point | DSM-5 | Australia |
| | BED | 2004-2004 | 14-14 | Female | 100·0 | 1800·0 | 501·4 | 703 | Point | DSM-5 | Australia |
| | BED | 2007-2007 | 17-17 | Female | 100·0 | 1400·0 | 443·1 | 703 | Point | DSM-5 | Australia |
| | BED | 2010-2010 | 20-20 | Female | 100·0 | 4100·0 | 747·9 | 703 | Point | DSM-5 | Australia |
| | BED | 2007-2007 | 17-17 | Male | 0·0 | 1200·0 | 417·6 | 680 | Point | DSM-5 | Australia |
| | BED | 2010-2010 | 20-20 | Male | 0·0 | 700·0 | 319·7 | 680 | Point | DSM-5 | Australia |
| | Bulimia Nervosa | 2004-2004 | 14-14 | Female | 100·0 | 2700·0 | 611·3 | 703 | Point | DSM-5 | Australia |
| | Bulimia Nervosa | 2007-2007 | 17-17 | Female | 100·0 | 8700·0 | 1063·0 | 703 | Point | DSM-5 | Australia |
| | Bulimia Nervosa | 2010-2010 | 20-20 | Female | 100·0 | 7900·0 | 1017·3 | 703 | Point | DSM-5 | Australia |
| | Bulimia Nervosa | 2004-2004 | 14-14 | Male | 0·0 | 400·0 | 242·1 | 680 | Point | DSM-5 | Australia |
| | Bulimia Nervosa | 2007-2007 | 17-17 | Male | 0·0 | 700·0 | 319·7 | 680 | Point | DSM-5 | Australia |
| | Bulimia Nervosa | 2010-2010 | 20-20 | Male | 0·0 | 1600·0 | 481·2 | 680 | Point | DSM-5 | Australia |

| | | | | | | | | | | | |
|---|---|---|---|---|---|---|---|---|---|---|---|
| | OSFED | 2004-2004 | 14-14 | Female | 100·0 | 3600·0 | 702·6 | 703 | Point | DSM-5 | Australia |
| | OSFED | 2007-2007 | 17-17 | Female | 100·0 | 4100·0 | 747·9 | 703 | Point | DSM-5 | Australia |
| | OSFED | 2010-2010 | 20-20 | Female | 100·0 | 2700·0 | 611·3 | 703 | Point | DSM-5 | Australia |
| | OSFED | 2004-2004 | 14-14 | Male | 0·0 | 700·0 | 319·7 | 680 | Point | DSM-5 | Australia |
| | OSFED | 2007-2007 | 17-17 | Male | 0·0 | 900·0 | 362·2 | 680 | Point | DSM-5 | Australia |
| | OSFED | 2010-2010 | 20-20 | Male | 0·0 | 600·0 | 296·2 | 680 | Point | DSM-5 | Australia |
| Beato-Fernández et al. (2004)[18] | Anorexia Nervosa | 2000-2001 | 13-15 | Female | 100·0 | 173·6 | 173·5 | 576 | Point | DSM-IV | Spain |
| | Anorexia Nervosa | 2000-2001 | 13-15 | Female | 100·0 | 173·6 | 173·5 | 576 | Point | DSM-IV | Spain |
| | Bulimia Nervosa | 2000-2001 | 13-15 | Female | 100·0 | 4861·1 | 896·1 | 576 | Point | DSM-IV | Spain |
| | OSFED + BED | 2000-2001 | 13-15 | Female | 100·0 | 4861·1 | 896·1 | 576 | Point | DSM-IV | Spain |
| Benjet et al. (2012)[19] | Anorexia Nervosa | 2005-2005 | 12-17 | Female | 100·0 | 200·0 | 100·0 | 1566 | Past year | DSM-IV | Mexico City |
| | Anorexia Nervosa | 2005-2005 | 12-17 | Female | 100·0 | 200·0 | 100·0 | 1566 | Past year | DSM-IV | Mexico City |
| | Anorexia Nervosa | 2005-2005 | 12-17 | Male | 0·0 | 100·0 | 100·0 | 1439 | Past year | DSM-IV | Mexico City |
| | BED | 2005-2005 | 12-17 | Female | 100·0 | 1100·0 | 200·0 | 1566 | Past year | DSM-IV | Mexico City |
| | BED | 2005-2005 | 12-17 | Male | 0·0 | 300·0 | 100·0 | 1439 | Past year | DSM-IV | Mexico City |
| | Bulimia Nervosa | 2005-2005 | 12-17 | Female | 100·0 | 900·0 | 300·0 | 1566 | Past year | DSM-IV | Mexico City |
| | Bulimia Nervosa | 2005-2005 | 12-17 | Male | 0·0 | 400·0 | 200·0 | 1439 | Past year | DSM-IV | Mexico City |
| Cotrufo et al. (1998)[20] | Anorexia Nervosa | 1996-1996 | 13-15 | Female | 100·0 | 404·0 | 285·1 | 495 | Point | DSM-IV | Italy |
| | Anorexia Nervosa | 1996-1996 | 13-15 | Female | 100·0 | 404·0 | 285·1 | 495 | Point | DSM-IV | Italy |
| | BED | 1996-1996 | 13-15 | Female | 100·0 | 404·0 | 285·1 | 495 | Point | DSM-IV | Italy |
| | BED | 1996-1996 | 17-19 | Female | 100·0 | 235·8 | 235·6 | 424 | Point | DSM-IV | Italy |
| | Bulimia Nervosa | 1996-1996 | 13-15 | Female | 100·0 | 1616·2 | 566·8 | 495 | Point | DSM-IV | Italy |
| | Bulimia Nervosa | 1996-1996 | 17-19 | Female | 100·0 | 3066·0 | 837·2 | 424 | Point | DSM-IV | Italy |
| | OSFED | 1996-1996 | 13-15 | Female | 100·0 | 3030·3 | 770·5 | 495 | Point | DSM-IV | Italy |
| | OSFED | 1996-1996 | 17-19 | Female | 100·0 | 5188·7 | 1077·1 | 424 | Point | DSM-IV | Italy |
| Duncan et al. (2017)[21] | Anorexia Nervosa | 2001-2003 | 18-99 | Male | 0·0 | 20·0 | 19·8 | 5091 | Past year | DSM-IV | United States of America |
| | Anorexia Nervosa | 2001-2003 | 18-99 | Male | 0·0 | 20·0 | 19·8 | 5091 | Past year | DSM-IV | United States of America |
| | BED | 2001-2003 | 18-99 | Female | 100·0 | 1430·0 | 139·5 | 7246 | Past year | DSM-IV | United States of America |
| | BED | 2001-2003 | 18-99 | Male | 0·0 | 670·0 | 114·3 | 5091 | Past year | DSM-IV | United States of America |

| | | | | | | | | | | | |
|---|---|---|---|---|---|---|---|---|---|---|---|
| | Bulimia Nervosa | 2001-2003 | 18-99 | Female | 100·0 | 670·0 | 95·8 | 7246 | Past year | DSM-IV | United States of America |
| | Bulimia Nervosa | 2001-2003 | 18-99 | Male | 0·0 | 190·0 | 61·0 | 5091 | Past year | DSM-IV | United States of America |
| Eapen et al. (2006)[22] | Anorexia Nervosa | 2003-2004 | 13-18 | Female | 100·0 | 202·0 | 201·8 | 495 | Point | DSM-IV | United Arab Emirates |
| | Bulimia Nervosa | 2003-2004 | 13-18 | Female | 100·0 | 202·0 | 201·8 | 495 | Point | DSM-IV | United Arab Emirates |
| Eddy et al. (2007)[23] | Anorexia Nervosa | 2005-2005 | 13-30 | Female | 100·0 | 1900·0 | 933·3 | 214 | Point | DSM-IV | United Republic of Tanzania |
| | Anorexia Nervosa | 2005-2005 | 13-30 | Female | 100·0 | 1900·0 | 933·3 | 214 | Point | DSM-IV | United Republic of Tanzania |
| | Bulimia Nervosa | 2005-2005 | 13-30 | Female | 100·0 | 467·3 | 466·2 | 214 | Point | DSM-IV | United Republic of Tanzania |
| | OSFED + BED | 2005-2005 | 13-30 | Female | 100·0 | 4700·0 | 1446·7 | 214 | Point | DSM-IV | United Republic of Tanzania |
| Faravelli et al. (2004)[24] | Anorexia Nervosa | 2001-2001 | 14-99 | Female | 100·0 | 300·0 | 153·1 | 1292 | Point | DSM-IV | Italy |
| | Bulimia Nervosa | 2001-2001 | 14-99 | Female | 100·0 | 400·0 | 153·1 | 1292 | Point | DSM-IV | Italy |
| Favaro et al. (2003)[25] | Anorexia Nervosa | 2001-2001 | 18-25 | Female | 100·0 | 300·0 | 178·6 | 934 | Point | DSM-IV | Italy |
| | Anorexia Nervosa | 2001-2001 | 18-25 | Female | 100·0 | 300·0 | 178·6 | 934 | Point | DSM-IV | Italy |
| | BED | 2001-2001 | 18-25 | Female | 100·0 | 100·0 | 76·5 | 934 | Point | DSM-IV | Italy |
| | Bulimia Nervosa | 2001-2001 | 18-25 | Female | 100·0 | 1800·0 | 433·7 | 934 | Point | DSM-IV | Italy |
| Fernández et al. (2007)[26] | Anorexia Nervosa | 2001-2002 | 12-21 | Female | 100·0 | 330·0 | 229·6 | 918 | Point | DSM-IV | Spain |
| | Anorexia Nervosa | 2001-2002 | 12-21 | Female | 100·0 | 330·0 | 229·6 | 918 | Point | DSM-IV | Spain |
| | Bulimia Nervosa | 2001-2002 | 12-21 | Female | 100·0 | 2290·0 | 492·4 | 918 | Point | DSM-IV | Spain |
| | Bulimia Nervosa | 2001-2002 | 12-21 | Male | 0·0 | 160·0 | 283·2 | 627 | Point | DSM-IV | Spain |
| | OSFED + BED | 2001-2002 | 12-21 | Female | 100·0 | 2720·0 | 538·3 | 918 | Point | DSM-IV | Spain |
| | OSFED + BED | 2001-2002 | 12-21 | Male | 0·0 | 480·0 | 339·3 | 627 | Point | DSM-IV | Spain |
| Fichter et al. (2005)[27] | Anorexia Nervosa | 1998-1998 | 12-21 | Female | 100·0 | 59·0 | 62·6 | 1506 | Point | DSM-IV | Greece |
| | Anorexia Nervosa | 1998-1998 | 12-21 | Female | 100·0 | 59·0 | 62·6 | 1506 | Point | DSM-IV | Greece |
| | Bulimia Nervosa | 1998-1998 | 12-21 | Female | 100·0 | 189·0 | 205·9 | 445 | Point | DSM-IV | Greece |
| | Bulimia Nervosa | 1998-1998 | 12-21 | Female | 100·0 | 118·0 | 88·5 | 1506 | Point | DSM-IV | Greece |
| | Bulimia Nervosa | 1998-1998 | 12-21 | Male | 0·0 | 68·0 | 69·3 | 1414 | Point | DSM-IV | Greece |
| | OSFED | 1998-1998 | 12-21 | Female | 100·0 | 1007·0 | 473·3 | 445 | Point | DSM-IV | Greece |
| | OSFED | 1998-1998 | 12-21 | Female | 100·0 | 1355·0 | 297·9 | 1506 | Point | DSM-IV | Greece |
| | OSFED | 1998-1998 | 12-21 | Male | 0·0 | 271·0 | 138·3 | 1414 | Point | DSM-IV | Greece |

| | | | | | | | | | | | |
|---|---|---|---|---|---|---|---|---|---|---|---|
| Gual et al. (2002)[28] | Anorexia Nervosa | 1997-1997 | 12-21 | Female | 100·0 | 300·0 | 127·6 | 2862 | Point | DSM-IV | Spain |
| | Anorexia Nervosa | 1997-1997 | 12-21 | Female | 100·0 | 300·0 | 127·6 | 2862 | Point | DSM-IV | Spain |
| | Bulimia Nervosa | 1997-1997 | 12-21 | Female | 100·0 | 800·0 | 178·6 | 2862 | Point | DSM-IV | Spain |
| | OSFED + BED | 1997-1997 | 12-21 | Female | 100·0 | 3100·0 | 331·6 | 2862 | Point | DSM-IV | Spain |
| Hay et al. (2015)[6] | Anorexia Nervosa | 2008-2009 | 15-96 | Both | 51·0 | 460·0 | 89·3 | 6041 | Point | DSM-5 | Australia |
| | Anorexia Nervosa | 2008-2009 | 15-96 | Both | 51·0 | 460·0 | 89·3 | 6041 | Point | DSM-5 | Australia |
| | BED | 2008-2009 | 15-96 | Both | 51·0 | 5580·0 | 295·9 | 6041 | Point | DSM-5 | Australia |
| | Bulimia Nervosa | 2008-2009 | 15-96 | Both | 51·0 | 660·0 | 104·6 | 6041 | Point | DSM-5 | Australia |
| | OSFED | 2008-2009 | 15-96 | Both | 51·0 | 8194·0 | 352·9 | 6041 | Point | DSM-5 | Australia |
| Hay et al. (2017)[7] | Bulimia Nervosa | 2015-2015 | 15-99 | Both | 50·0 | 1200·0 | 204·1 | 3005 | Point | DSM-5 | Australia |
| | OSFED | 2015-2015 | 15-99 | Both | 50·0 | 3200·0 | 331·6 | 3005 | Point | DSM-5 | Australia |
| Huang et al. (2019)[29] | Anorexia Nervosa | 2013-2015 | 18-99 | Male | 0·0 | 7·8 | 2·3 | 12776 | Past year | DSM-IV | China |
| | Bulimia Nervosa | 2013-2015 | 18-99 | Male | 0·0 | 23·5 | 9·9 | 12776 | Past year | DSM-IV | China |
| Huon et al. (2002)[30] | Anorexia Nervosa | 2000-2000 | 12-19 | Female | 100·0 | 200·0 | 126·6 | 1246 | Point | DSM-IV | China |
| | Anorexia Nervosa | 2000-2000 | 12-19 | Female | 100·0 | 200·0 | 126·6 | 1246 | Point | DSM-IV | China |
| | BED | 2000-2000 | 12-19 | Female | 100·0 | 561·8 | 211·7 | 1246 | Point | DSM-IV | China |
| | OSFED | 2000-2000 | 12-19 | Female | 100·0 | 1765·7 | 373·1 | 1246 | Point | DSM-IV | China |
| Isomaa et al. (2009)[31] | Anorexia Nervosa | 2005-2005 | 15-15 | Both | 47·6 | 2174·1 | 597·9 | 595 | Point | DSM-IV | Finland |
| | Anorexia Nervosa | 2005-2005 | 15-15 | Both | 47·6 | 2174·1 | 597·9 | 595 | Point | DSM-IV | Finland |
| | Anorexia Nervosa | 2007-2007 | 18-18 | Both | 50·6 | 4500·0 | 964·5 | 462 | Point | DSM-IV | Finland |
| | Bulimia Nervosa | 2005-2005 | 15-15 | Both | 47·6 | 4615·7 | 860·2 | 595 | Point | DSM-IV | Finland |
| | Bulimia Nervosa | 2007-2007 | 18-18 | Both | 50·6 | 2050·0 | 659·3 | 462 | Point | DSM-IV | Finland |
| | OSFED + BED | 2005-2005 | 15-15 | Both | 47·6 | 2137·5 | 592·9 | 595 | Point | DSM-IV | Finland |
| | OSFED + BED | 2007-2007 | 18-18 | Both | 50·6 | 2950·0 | 787·2 | 462 | Point | DSM-IV | Finland |
| Jacobi et al. (2015)[32] | Anorexia Nervosa | 2008-2011 | 18-79 | Female | 100·0 | 1200·0 | 280·6 | 2778 | Past year | DSM-IV | Germany |
| | Anorexia Nervosa | 2008-2011 | 18-79 | Female | 100·0 | 1200·0 | 280·6 | 2778 | Past year | DSM-IV | Germany |
| | Anorexia Nervosa | 2008-2011 | 18-79 | Male | 0·0 | 300·0 | 153·1 | 2540 | Past year | DSM-IV | Germany |
| | Bulimia Nervosa | 2008-2011 | 18-79 | Female | 100·0 | 300·0 | 76·5 | 2778 | Past year | DSM-IV | Germany |
| | Bulimia Nervosa | 2008-2011 | 18-79 | Male | 0·0 | 100·0 | 76·5 | 2540 | Past year | DSM-IV | Germany |
| | OSFED + BED | 2008-2011 | 18-79 | Female | 100·0 | 100·0 | 102·0 | 2778 | Past year | DSM-IV | Germany |

| | | | | | | | | | | | |
|---|---|---|---|---|---|---|---|---|---|---|---|
| | OSFED + BED | 2008-2011 | 18-79 | Male | 0·0 | 100·0 | 51·0 | 2540 | Past year | DSM-IV | Germany |
| Karg et al. (2012)[33] | Anorexia Nervosa | 2008-2012 | 18-99 | Female | 100·0 | 8·5 | 17·0 | 2936 | Past year | DSM-IV | United States of America |
| | Anorexia Nervosa | 2008-2012 | 18-99 | Male | 0·0 | 1·8 | 8·2 | 2715 | Past year | DSM-IV | United States of America |
| | Bulimia Nervosa | 2008-2012 | 18-99 | Female | 100·0 | 41·5 | 37·6 | 2936 | Past year | DSM-IV | United States of America |
| | Bulimia Nervosa | 2008-2012 | 18-99 | Male | 0·0 | 8·2 | 17·4 | 2715 | Past year | DSM-IV | United States of America |
| Keqing et al. (2008)[34] | Anorexia Nervosa | 2004-2005 | 18-95 | Female | 100·0 | 19·0 | 13·5 | 10373 | Point | DSM-IV | Hebei |
| | Anorexia Nervosa | 2004-2005 | 18-95 | Female | 100·0 | 19·0 | 13·5 | 10373 | Point | DSM-IV | Hebei |
| | BED | 2004-2005 | 18-95 | Female | 100·0 | 10·0 | 9·8 | 10373 | Point | DSM-IV | Hebei |
| | Bulimia Nervosa | 2004-2005 | 18-95 | Female | 100·0 | 10·0 | 9·8 | 10373 | Point | DSM-IV | Hebei |
| Kessler et al. (2013)[35] | BED | 2001-2002 | 18-65 | Both | 50·0 | 500·0 | 200·0 | 1236 | Past year | DSM-IV | Mexico |
| | BED | 2003-2003 | 18-65 | Both | 50·0 | 300·0 | 100·0 | 1217 | Past year | DSM-IV | Colombia |
| | Bulimia Nervosa | 2001-2002 | 18-65 | Both | 50·0 | 300·0 | 100·0 | 1236 | Past year | DSM-IV | Mexico |
| | Bulimia Nervosa | 2003-2003 | 18-65 | Both | 50·0 | 200·0 | 100·0 | 1217 | Past year | DSM-IV | Colombia |
| Khaleghi et al. (2018)[36] | Anorexia Nervosa | 2017-2017 | 6-18 | Both | 48·6 | 100·0 | 5·1 | 2095 | Point | DSM-IV | Iran (Islamic Republic of) |
| | Bulimia Nervosa | 2017-2017 | 6-18 | Both | 48·6 | 300·0 | 130·1 | 2095 | Point | DSM-IV | Iran (Islamic Republic of) |
| Kim-Cohen et al. (2003)[37] | Anorexia Nervosa | 2000-2002 | 26-26 | Female | 100·0 | 1531·9 | 568·9 | 466 | Point | DSM-IV | New Zealand |
| | Anorexia Nervosa | 2000-2002 | 26-26 | Male | 0·0 | 566·4 | 334·0 | 505 | Point | DSM-IV | New Zealand |
| | Bulimia Nervosa | 2000-2002 | 26-26 | Female | 100·0 | 1993·2 | 647·4 | 466 | Point | DSM-IV | New Zealand |
| | Bulimia Nervosa | 2000-2002 | 26-26 | Male | 0·0 | 734·8 | 380·1 | 505 | Point | DSM-IV | New Zealand |
| Kinzl et al. (1999)[38] | BED | 1997-1997 | 18-88 | Male | 0·0 | 800·0 | 281·7 | 1000 | Point | EDE | Austria |
| | Bulimia | 1997-1997 | 18-88 | Male | 0·0 | 500·0 | 223·0 | 1000 | Point | EDE | Austria |
| | OSFED | 1997-1997 | 18-88 | Male | 0·0 | 9400·0 | 922·8 | 1000 | Point | EDE | Austria |
| La Maison et al. (2018)[39] | Anorexia Nervosa | 2015-2015 | 11-11 | Both | 48·4 | 140·4 | 63·8 | 3562 | Point | DSM-5 | Rio Grande do Sul |
| | Anorexia Nervosa | 2015-2015 | 11-11 | Both | 48·4 | 140·4 | 63·8 | 3562 | Point | DSM-5 | Rio Grande do Sul |
| | BED | 2015-2015 | 11-11 | Both | 48·4 | 336·9 | 102·0 | 3562 | Point | DSM-5 | Rio Grande do Sul |
| | OSFED | 2015-2015 | 11-11 | Both | 48·4 | 28·1 | 51·0 | 3562 | Point | DSM-5 | Rio Grande do Sul |
| Lähteenmäki et al. (2014)[40] | Anorexia Nervosa | 2003-2005 | 20-35 | Female | 100·0 | 300·0 | 561·2 | 333 | Point | DSM-IV | Finland |
| | Anorexia Nervosa | 2003-2005 | 20-35 | Female | 100·0 | 300·0 | 561·2 | 333 | Point | DSM-IV | Finland |

| | | | | | | | | | | | |
|---|---|---|---|---|---|---|---|---|---|---|---|
| | Bulimia Nervosa | 2003-2005 | 20-35 | Female | 100·0 | 1400·0 | 714·3 | 429 | Point | DSM-IV | Finland |
| | OSFED + BED | 2003-2005 | 20-35 | Female | 100·0 | 500·0 | 484·7 | 400 | Point | DSM-IV | Finland |
| Ma et al. (2008)[41] | Anorexia Nervosa | 2004-2005 | 18-95 | Both | 49·3 | 33·0 | 17·9 | 9021 | Point | DSM-IV | Hebei |
| | BED | 2004-2005 | 18-95 | Both | 49·3 | 11·0 | 8·4 | 9021 | Point | DSM-IV | Hebei |
| Machado et al. (2007)[42] | Anorexia Nervosa | 2005-2005 | 12-23 | Female | 100·0 | 390·0 | 140·3 | 2028 | Point | DSM-IV | Portugal |
| | Anorexia Nervosa | 2005-2005 | 12-23 | Female | 100·0 | 390·0 | 140·3 | 2028 | Point | DSM-IV | Portugal |
| | Bulimia Nervosa | 2005-2005 | 12-23 | Female | 100·0 | 300·0 | 119·9 | 2028 | Point | DSM-IV | Portugal |
| | OSFED + BED | 2005-2005 | 12-23 | Female | 100·0 | 2370·0 | 341·8 | 2028 | Point | DSM-IV | Portugal |
| Mangweth-Matzek et al. (2014)[43] | BED | 2011-2013 | 40-60 | Female | 100·0 | 1538·5 | 460·3 | 715 | Point | DSM-IV | Austria |
| | BED | 2011-2013 | 40-60 | Female | 100·0 | 1538·5 | 460·3 | 715 | Point | DSM-IV | Austria |
| | Bulimia Nervosa | 2011-2013 | 40-60 | Female | 100·0 | 1398·6 | 439·2 | 715 | Point | DSM-IV | Austria |
| | OSFED + BED | 2011-2013 | 40-60 | Female | 100·0 | 3216·8 | 659·9 | 715 | Point | DSM-IV | Austria |
| Mangweth-Matzek et al. (2006)[44] | Anorexia Nervosa | 2004-2004 | 60-70 | Female | 100·0 | 210·5 | 210·3 | 475 | Point | DSM-IV | Austria |
| | Anorexia Nervosa | 2004-2004 | 60-70 | Female | 100·0 | 210·5 | 210·3 | 475 | Point | DSM-IV | Austria |
| | BED | 2004-2004 | 60-70 | Female | 100·0 | 1052·6 | 468·3 | 475 | Point | DSM-IV | Austria |
| | Bulimia Nervosa | 2004-2004 | 60-70 | Female | 100·0 | 421·1 | 297·1 | 475 | Point | DSM-IV | Austria |
| | OSFED + BED | 2004-2004 | 60-70 | Female | 100·0 | 3157·9 | 802·4 | 475 | Point | DSM-IV | Austria |
| Merikangas et al. (2010)[45] | Anorexia Nervosa | 2001-2004 | 8-15 | Both | 51·0 | 100·0 | 57·3 | 3042 | Point | DSM-IV | United States of America |
| | Bulimia Nervosa | 2001-2004 | 8-15 | Both | 51·0 | 100·0 | 57·3 | 3042 | Point | DSM-IV | United States of America |
| Michel et al. (2018)[46] | Anorexia Nervosa | 2011-2014 | 16-40 | Both | 46·1 | 400·0 | 121·9 | 2683 | Point | DSM-IV | Switzerland |
| | Bulimia Nervosa | 2011-2014 | 16-40 | Both | 46·1 | 100·0 | 61·0 | 2683 | Point | DSM-IV | Switzerland |
| Mohammadi et al. (2017)[47] | Anorexia Nervosa | 2013-2015 | 6-18 | Female | 100·0 | 190·0 | 25·5 | 4737 | Point | DSM-IV | Iran (Islamic Republic of) |
| | Anorexia Nervosa | 2013-2015 | 6-18 | Male | 0·0 | 120·0 | 25·5 | 5000 | Point | DSM-IV | Iran (Islamic Republic of) |
| | Bulimia Nervosa | 2013-2015 | 6-18 | Female | 100·0 | 100·0 | 25·5 | 4737 | Point | DSM-IV | Iran (Islamic Republic of) |
| | Bulimia Nervosa | 2013-2015 | 6-18 | Male | 0·0 | 120·0 | 25·5 | 5000 | Point | DSM-IV | Iran (Islamic Republic of) |
| Mohler-Kuo et al. (2016)[48] | Anorexia | 2010-2010 | 15-60 | Female | 100·0 | 70·0 | 43·4 | 5615 | Past year | CIDI | Switzerland |
| | Anorexia | 2010-2010 | 15-60 | Female | 100·0 | 70·0 | 43·4 | 5615 | Past year | CIDI | Switzerland |
| | Anorexia | 2010-2010 | 15-60 | Male | 0·0 | 30·0 | 50·0 | 4423 | Past year | CIDI | Switzerland |

| | | | | | | | | | | | |
|---|---|---|---|---|---|---|---|---|---|---|---|
| | BED | 2010-2010 | 15-60 | Female | 100·0 | 900·0 | 153·1 | 5615 | Past year | CIDI | Switzerland |
| | BED | 2010-2010 | 15-60 | Male | 0·0 | 300·0 | 127·6 | 4423 | Past year | CIDI | Switzerland |
| | Bulimia | 2010-2010 | 15-60 | Female | 100·0 | 600·0 | 102·0 | 5615 | Past year | CIDI | Switzerland |
| | Bulimia | 2010-2010 | 15-60 | Male | 0·0 | 500·0 | 51·0 | 4423 | Past year | CIDI | Switzerland |
| Mousa et al. (2010)[49] | BED | 2008-2008 | 10-16 | Female | 100·0 | 1800·0 | 736·3 | 326 | Point | DSM-IV | Jordan |
| | BED | 2008-2008 | 10-16 | Female | 100·0 | 1800·0 | 736·3 | 326 | Point | DSM-IV | Jordan |
| | Bulimia Nervosa | 2008-2008 | 10-16 | Female | 100·0 | 600·0 | 427·7 | 326 | Point | DSM-IV | Jordan |
| | OSFED | 2008-2008 | 10-16 | Female | 100·0 | 31000·0 | 2561·5 | 326 | Point | DSM-IV | Jordan |
| Qu et al. (2015)[50] | Anorexia Nervosa | 2013-2014 | 6-16 | Both | 49·6 | 12000·0 | 2296·0 | 19711 | Point | DSM-IV | Sichuan |
| | Bulimia Nervosa | 2013-2014 | 6-16 | Both | 49·6 | 80000·0 | 6377·7 | 19711 | Point | DSM-IV | Sichuan |
| Rosenvinge et al. (1999)[51] | Anorexia | 1996-1998 | 15-15 | Both | 100·0 | 431·0 | 304·1 | 464 | Point | DSED | Norway |
| | Anorexia | 1996-1998 | 15-15 | Both | 100·0 | 431·0 | 304·1 | 464 | Point | DSED | Norway |
| | BED | 1996-1998 | 15-15 | Both | 100·0 | 1508·6 | 565·9 | 464 | Point | DSED | Norway |
| | Bulimia | 1996-1998 | 15-15 | Both | 100·0 | 1077·6 | 479·3 | 464 | Point | DSED | Norway |
| Ruiz-Lazaro et al. (2005)[52] | Anorexia Nervosa | 1999-1999 | 12-18 | Female | 100·0 | 140·0 | 102·0 | 2193 | Point | DSM-IV | Spain |
| | Anorexia Nervosa | 1999-1999 | 12-18 | Female | 100·0 | 140·0 | 102·0 | 2193 | Point | DSM-IV | Spain |
| | Bulimia Nervosa | 1999-1999 | 12-18 | Female | 100·0 | 550·0 | 153·1 | 2193 | Point | DSM-IV | Spain |
| | OSFED + BED | 1999-1999 | 12-18 | Female | 100·0 | 3830·0 | 943·9 | 2193 | Point | DSM-IV | Spain |
| Salum et al. (2015)[53] | Anorexia Nervosa | 2012-2014 | 6-12 | Both | 46·9 | 39·8 | 39·8 | 2512 | Point | DSM-IV | Brazil |
| | OSFED + BED | 2012-2014 | 6-12 | Both | 46·9 | 400·0 | 125·9 | 2512 | Point | DSM-IV | Brazil |
| Sancho et al. (2008)[54] | Anorexia Nervosa | 2006-2006 | 9-13 | Both | 51·4 | 374·3 | 167·1 | 1336 | Point | DSM-IV | Spain |
| | Bulimia Nervosa | 2006-2006 | 9-13 | Both | 51·4 | 224·6 | 129·5 | 1336 | Point | DSM-IV | Spain |
| Semiz et al. (2013)[55] | Bulimia Nervosa | 2010-2010 | 18-44 | Female | 100·0 | 1296·3 | 486·8 | 540 | Point | DSM-IV | Turkey |
| | OSFED + BED | 2010-2010 | 18-44 | Female | 100·0 | 1481·5 | 519·9 | 540 | Point | DSM-IV | Turkey |
| Smink et al. (2014)[56] | Anorexia Nervosa | 2010-2010 | 19-19 | Female | 100·0 | 1200·0 | 382·7 | 861 | Point | DSM-5 | Netherlands |
| | Anorexia Nervosa | 2010-2010 | 19-19 | Female | 100·0 | 1200·0 | 382·7 | 861 | Point | DSM-5 | Netherlands |
| | Anorexia Nervosa | 2010-2010 | 19-19 | Male | 0·0 | 100·0 | 204·1 | 736 | Point | DSM-5 | Netherlands |
| | BED | 2010-2010 | 19-19 | Female | 100·0 | 1600·0 | 459·2 | 861 | Point | DSM-5 | Netherlands |
| | BED | 2010-2010 | 19-19 | Male | 0·0 | 300·0 | 255·1 | 736 | Point | DSM-5 | Netherlands |
| | Bulimia Nervosa | 2010-2010 | 19-19 | Female | 100·0 | 600·0 | 280·6 | 861 | Point | DSM-5 | Netherlands |

| | | | | | | | | | | | |
|---|---|---|---|---|---|---|---|---|---|---|---|
| | Bulimia Nervosa | 2010-2010 | 19-19 | Male | 0·0 | 100·0 | 204·1 | 736 | Point | DSM-5 | Netherlands |
| | OSFED | 2010-2010 | 19-19 | Female | 100·0 | 300·0 | 229·6 | 861 | Point | DSM-5 | Netherlands |
| Solmi et al. (2016)[57] | BED | 2008-2010 | 16-90 | Both | 66·0 | 3600·0 | 1938·8 | 1698 | Past year | SCID | England |
| | BED | 2008-2010 | 16-90 | Both | 66·0 | 3600·0 | 1938·8 | 1698 | Past year | SCID | England |
| | Bulimia | 2008-2010 | 16-90 | Both | 66·0 | 800·0 | 382·7 | 1698 | Past year | SCID | England |
| | OSFED | 2008-2010 | 16-90 | Both | 66·0 | 3000·0 | 1516·3 | 1698 | Past year | SCID | England |
| Stice et al. (2013)[11] | Anorexia | 2003-2013 | 16-19 | Female | 100·0 | 201·6 | 201·4 | 496 | Past year | EDDI | United States of America |
| | Anorexia | 2003-2013 | 16-19 | Female | 100·0 | 201·6 | 201·4 | 496 | Past year | EDDI | United States of America |
| | Anorexia | 2003-2013 | 17-20 | Female | 100·0 | 201·6 | 201·4 | 496 | Past year | EDDI | United States of America |
| | Anorexia | 2003-2013 | 18-21 | Female | 100·0 | 403·2 | 284·5 | 496 | Past year | EDDI | United States of America |
| | Anorexia | 2003-2013 | 19-22 | Female | 100·0 | 604·8 | 348·1 | 496 | Past year | EDDI | United States of America |
| | BED | 2003-2013 | 12-15 | Female | 100·0 | 403·2 | 284·5 | 496 | Past year | EDDI | United States of America |
| | BED | 2003-2013 | 13-16 | Female | 100·0 | 403·2 | 284·5 | 496 | Past year | EDDI | United States of America |
| | BED | 2003-2013 | 14-17 | Female | 100·0 | 201·6 | 201·4 | 496 | Past year | EDDI | United States of America |
| | BED | 2003-2013 | 15-18 | Female | 100·0 | 201·6 | 201·4 | 496 | Past year | EDDI | United States of America |
| | BED | 2003-2013 | 16-19 | Female | 100·0 | 604·8 | 348·1 | 496 | Past year | EDDI | United States of America |
| | BED | 2003-2013 | 17-20 | Female | 100·0 | 604·8 | 348·1 | 496 | Past year | EDDI | United States of America |
| | BED | 2003-2013 | 18-21 | Female | 100·0 | 1411·3 | 529·6 | 496 | Past year | EDDI | United States of America |
| | BED | 2003-2013 | 19-22 | Female | 100·0 | 604·8 | 348·1 | 496 | Past year | EDDI | United States of America |
| | Bulimia | 2003-2013 | 12-15 | Female | 100·0 | 403·2 | 284·5 | 496 | Past year | EDDI | United States of America |
| | Bulimia | 2003-2013 | 13-16 | Female | 100·0 | 201·6 | 201·4 | 496 | Past year | EDDI | United States of America |
| | Bulimia | 2003-2013 | 14-17 | Female | 100·0 | 201·6 | 201·4 | 496 | Past year | EDDI | United States of America |
| | Bulimia | 2003-2013 | 15-18 | Female | 100·0 | 201·6 | 201·4 | 496 | Past year | EDDI | United States of America |
| | Bulimia | 2003-2013 | 16-19 | Female | 100·0 | 403·2 | 284·5 | 496 | Past year | EDDI | United States of America |
| | Bulimia | 2003-2013 | 17-20 | Female | 100·0 | 1008·1 | 448·5 | 496 | Past year | EDDI | United States of America |

| | | | | | | | | | | | |
|---|---|---|---|---|---|---|---|---|---|---|---|
| | Bulimia | 2003-2013 | 18-21 | Female | 100·0 | 806·5 | 401·6 | 496 | Past year | EDDI | United States of America |
| | Bulimia | 2003-2013 | 19-22 | Female | 100·0 | 604·8 | 348·1 | 496 | Past year | EDDI | United States of America |
| Swanson et al. (2011)[58] | Anorexia Nervosa | 2001-2004 | 13-18 | Female | 100·0 | 100·0 | 60·0 | 5173 | Past year | DSM-IV | Minnesota |
| | Anorexia Nervosa | 2001-2004 | 13-18 | Female | 100·0 | 100·0 | 60·0 | 5173 | Past year | DSM-IV | Minnesota |
| | Anorexia Nervosa | 2001-2004 | 13-18 | Male | 0·0 | 200·0 | 80·0 | 4950 | Past year | DSM-IV | Minnesota |
| | BED | 2001-2004 | 13-18 | Female | 100·0 | 1400·0 | 330·0 | 5173 | Past year | DSM-IV | Minnesota |
| | BED | 2001-2004 | 13-18 | Male | 0·0 | 400·0 | 90·0 | 4950 | Past year | DSM-IV | Minnesota |
| | Bulimia Nervosa | 2001-2004 | 13-18 | Female | 100·0 | 900·0 | 170·0 | 5173 | Past year | DSM-IV | Minnesota |
| | Bulimia Nervosa | 2001-2004 | 13-18 | Male | 0·0 | 300·0 | 220·0 | 4950 | Past year | DSM-IV | Minnesota |
| Szumska et al. (2005)[59] | Anorexia Nervosa | 1998-1998 | 15-24 | Female | 100·0 | 30·0 | 29·8 | 3386 | Point | DSM-IV | Hungary |
| | Bulimia Nervosa | 1998-1998 | 15-24 | Female | 100·0 | 410·0 | 109·8 | 3386 | Point | DSM-IV | Hungary |
| Vardar & Erzengin (2011) | Anorexia Nervosa | 2009-2009 | 15-17 | Female | 100·0 | 68·0 | 68·1 | 1464 | Point | DSM-IV | Turkey |
| | Anorexia Nervosa | 2009-2009 | 15-17 | Female | 100·0 | 68·0 | 68·1 | 1464 | Point | DSM-IV | Turkey |
| | BED | 2009-2009 | 15-17 | Female | 100·0 | 1430·0 | 310·3 | 1464 | Point | DSM-IV | Turkey |
| | BED | 2009-2009 | 15-17 | Male | 0·0 | 550·0 | 194·7 | 1443 | Point | DSM-IV | Turkey |
| | Bulimia Nervosa | 2009-2009 | 15-17 | Female | 100·0 | 1570·0 | 324·9 | 1464 | Point | DSM-IV | Turkey |
| | OSFED + BED | 2009-2009 | 15-17 | Female | 100·0 | 2390·0 | 399·2 | 1464 | Point | DSM-IV | Turkey |
| | OSFED + BED | 2009-2009 | 15-17 | Male | 0·0 | 620·0 | 206·6 | 1443 | Point | DSM-IV | Turkey |
| Wagner et al. (2017)[60] | Anorexia Nervosa | 2013-2015 | 10-18 | Both | 56·5 | 1010·0 | 459·2 | 3579 | Point | DSM-5 | Austria |
| | Anorexia Nervosa | 2013-2015 | 10-18 | Both | 56·5 | 1010·0 | 459·2 | 3579 | Point | DSM-5 | Austria |
| | BED | 2013-2015 | 10-18 | Both | 56·5 | 120·0 | 153·1 | 3579 | Point | DSM-5 | Austria |
| | Bulimia Nervosa | 2013-2015 | 10-18 | Both | 56·5 | 170·0 | 204·1 | 3579 | Point | DSM-5 | Austria |
| | OSFED | 2013-2015 | 10-18 | Both | 56·5 | 300·0 | 255·1 | 3579 | Point | DSM-5 | Austria |
| Wells et al. (2006)[61] | Anorexia Nervosa | 2003-2004 | 16-99 | Both | 59·4 | 100·0 | 25·5 | 7435 | Past year | DSM-IV | New Zealand |
| | Bulimia Nervosa | 2003-2004 | 16-99 | Both | 59·4 | 400·0 | 76·5 | 7435 | Past year | DSM-IV | New Zealand |
| West et al. (2003)[62] | Anorexia Nervosa | 1999-1999 | 15-15 | Female | 100·0 | 100·0 | 109·4 | 834 | Point | DSM-IV | Scotland |
| | Bulimia Nervosa | 1999-1999 | 15-15 | Female | 100·0 | 100·0 | 109·4 | 834 | Point | DSM-IV | Scotland |
| Wittchen et al. (1998)[63] | Anorexia Nervosa | 1995-1995 | 14-24 | Female | 100·0 | 300·0 | 139·9 | 1528 | Past year | DSM-IV | Germany |
| | Anorexia Nervosa | 1995-1995 | 14-24 | Female | 100·0 | 300·0 | 139·9 | 1528 | Past year | DSM-IV | Germany |

| | | | | | | | | | | | |
|---|---|---|---|---|---|---|---|---|---|---|---|
| | Bulimia Nervosa | 1995-1995 | 14-24 | Female | 100·0 | 700·0 | 213·3 | 1528 | Past year | DSM-IV | Germany |
| | OSFED + BED | 1995-1995 | 14-24 | Female | 100·0 | 1400·0 | 300·6 | 1528 | Past year | DSM-IV | Germany |

BED = Binge-eating disorder, DSM = Diagnostic and Statistical Manual of Mental Disorder, OSFED = Other specified feeding and eating disorder, SE = Standard error. Estimates marked OSFED + BED reported prevalence for Eating disorder not otherwise specified (EDNOS) and did not isolate the prevalence of BED. Table excludes estimates of zero prevalence.

## References


1. Erskine HE, Whiteford HA. Epidemiology of binge eating disorder. *Current opinion in psychiatry* 2018; **31**(6): 462-70.
2. Lindvall Dahlgren C, Wisting L, Rø Ø. Feeding and eating disorders in the DSM-5 era: a systematic review of prevalence rates in non-clinical male and female samples. *J Eat Disord* 2017; **5**: 56.
3. GBD 2019 Diseases and Injuries Collaborators. Global burden of 369 diseases and injuries in 204 countries and territories, 1990–2019: a systematic analysis for the Global Burden of Disease Study 2019. . *The Lancet* 2020; **396**(10258): 1204-22.
4. Salomon JA, Haagsma JA, Davis A, et al. Disability weights for the Global Burden of Disease 2013 study. *The Lancet Global health* 2015; **3**(11): e712-23.
5. Allen KL, Byrne SM, Oddy WH, Crosby RD. DSM–IV–TR and DSM-5 eating disorders in adolescents: Prevalence, stability, and psychosocial correlates in a population-based sample of male and female adolescents. *Journal of Abnormal Psychology* 2013; **122**(3): 720-32.
6. Hay P, Girosi F, Mond J. Prevalence and sociodemographic correlates of DSM-5 eating disorders in the Australian population. *Journal of Eating Disorders* 2015; **3**(1): 19.
7. Hay P, Mitchison D, Collado AEL, González-Chica DA, Stocks N, Touyz S. Burden and health-related quality of life of eating disorders, including Avoidant/Restrictive Food Intake Disorder (ARFID), in the Australian population. *Journal of Eating Disorders* 2017; **5**(1): 21.
8. Hammerle F, Huss M, Ernst V, Bürger A. Thinking dimensional: prevalence of DSM-5 early adolescent full syndrome, partial and subthreshold eating disorders in a cross-sectional survey in German schools. *BMJ open* 2016; **6**(5): e010843.
9. Mitchison D, Mond J, Bussey K, et al. DSM-5 full syndrome, other specified, and unspecified eating disorders in Australian adolescents: prevalence and clinical significance. *Psychological medicine* 2020; **50**(6): 981-90.
10. Micali N, Solmi F, Horton NJ, et al. Adolescent Eating Disorders Predict Psychiatric, High-Risk Behaviors and Weight Outcomes in Young Adulthood. *Journal of the American Academy of Child and Adolescent Psychiatry* 2015; **54**(8): 652-9.e1.
11. Stice E, Marti CN, Rohde P. Prevalence, incidence, impairment, and course of the proposed DSM-5 eating disorder diagnoses in an 8-year prospective community study of young women. *Journal of Abnormal Psychology* 2013; **122**(2): 445-57.
12. American Psychiatric Association. Diagnostic and statistical manual of mental disorders: DSM-5. 18th ed. Arlington, VA: American Psychiatric Association; 2013.
13. American Psychiatric Association. Diagnostic and statistical manual of mental disorders: DSM-IV-TR. Washington, DC: American Psychiatric Association; 2000.
14. Zheng P, Aravkin AY, Barber R, Sorensen RJD, Murray CJL. Trimmed Constrained Mixed Effects Models: Formulations and Algorithms. *arXiv:190910700 [math, stat]* 2019: http://arxiv.org/abs/1909.10700 (accessed 28th September 2020).
15. Aalto-Setälä T, Marttunen M, Tuulio-Henriksson A, Poikolainen K, Lönnqvist J. One-month prevalence of depression and other DSM-IV disorders among young adults. *Psychological medicine* 2001; **31**(5): 791-801.
16. Ackard DM, Fulkerson JA, Neumark-Sztainer D. Prevalence and utility of DSM-IV eating disorder diagnostic criteria among youth. *The International journal of eating disorders* 2007; **40**(5): 409-17.
17. Alatiq Y, Alshalan M, Almodayfer O. Prevalence of Psychiatric Disorders among Saudi Adolescent Girls in a Riyadh City High School. *The Arab Journal of Psychiatry* 2017; **28**.

18. Beato-Fernández L, Rodríguez-Cano T, Belmonte-Llario A, Martínez-Delgado C. Risk factors for eating disorders in adolescents. A Spanish community-based longitudinal study. *European child & adolescent psychiatry* 2004; **13**(5): 287-94.
19. Benjet C, Mendez E, Borges G, Medina-Mora M. Epidemiology of eating disorders in a representative sample of adolescents. *Salud Mental* 2012; **35**: 483-90.
20. Cotrufo P, Barretta V, Monteleone P, Maj M. Full-syndrome, partial-syndrome and subclinical eating disorders: an epidemiological study of female students in Southern Italy. *Acta psychiatrica Scandinavica* 1998; **98**(2): 112-5.
21. Duncan AE, Ziobrowski HN, Nicol G. The Prevalence of Past 12-Month and Lifetime DSM-IV Eating Disorders by BMI Category in US Men and Women. *European Eating Disorders Review* 2017; **25**(3): 165-71.
22. Eapen V, Mabrouk AA, Bin-Othman S. Disordered eating attitudes and symptomatology among adolescent girls in the United Arab Emirates. *Eating behaviors* 2006; **7**(1): 53-60.
23. Eddy KT, Hennessey M, Thompson-Brenner H. Eating pathology in East African women: the role of media exposure and globalization. *The Journal of nervous and mental disease* 2007; **195**(3): 196-202.
24. Faravelli C, Abrardi L, Bartolozzi D, et al. The Sesto Fiorentino study: point and one-year prevalences of psychiatric disorders in an Italian community sample using clinical interviewers. *Psychotherapy and psychosomatics* 2004; **73**(4): 226-34.
25. Favaro A, Ferrara S, Santonastaso P. The spectrum of eating disorders in young women: a prevalence study in a general population sample. *Psychosomatic medicine* 2003; **65**(4): 701-8.
26. Peláez Fernández MA, Labrador FJ, Raich RM. Prevalence of eating disorders among adolescent and young adult scholastic population in the region of Madrid (Spain). *Journal of psychosomatic research* 2007; **62**(6): 681-90.
27. Fichter MM, Quadflieg N, Georgopoulou E, Xepapadakos F, Fthenakis EW. Time trends in eating disturbances in young Greek migrants. *The International journal of eating disorders* 2005; **38**(4): 310-22.
28. Gual P, Pérez-Gaspar M, Martínez-González MA, Lahortiga F, de Irala-Estévez J, Cervera-Enguix S. Self-esteem, personality, and eating disorders: baseline assessment of a prospective population-based cohort. *The International journal of eating disorders* 2002; **31**(3): 261-73.
29. Huang Y, Wang Y, Wang H, et al. Prevalence of mental disorders in China: a cross-sectional epidemiological study. *The Lancet Psychiatry* 2019; **6**(3): 211-24.
30. Huon GF, Mingyi Q, Oliver K, Xiao G. A large-scale survey of eating disorder symptomatology among female adolescents in the People's Republic of China. *The International journal of eating disorders* 2002; **32**(2): 192-205.
31. Isomaa R, Isomaa AL, Marttunen M, Kaltiala-Heino R, Björkqvist K. The prevalence, incidence and development of eating disorders in Finnish adolescents: a two-step 3-year follow-up study. *European eating disorders review : the journal of the Eating Disorders Association* 2009; **17**(3): 199-207.
32. Jacobi F, Höfler M, Strehle J, et al. Twelve-months prevalence of mental disorders in the German Health Interview and Examination Survey for Adults - Mental Health Module (DEGS1-MH): a methodological addendum and correction. *International journal of methods in psychiatric research* 2015; **24**(4): 305-13.
33. Karg RS, Bose J, Batts KR, et al. Past Year Mental Disorders among Adults in the United States: Results from the 2008–2012 Mental Health Surveillance Study. CBHSQ Data Review. Rockville (MD): Substance Abuse and Mental Health Services Administration (US); 2012: 1-19.

34. Keqing L, Ze C, Lijun C, et al. Epidemiological survey of mental disorders in the people aged 18 and older in Hebei Province. *Asian journal of psychiatry* 2008; **1**(2): 51-5.
35. Kessler RC, Berglund PA, Chiu WT, et al. The prevalence and correlates of binge eating disorder in the World Health Organization World Mental Health Surveys. *Biological psychiatry* 2013; **73**(9): 904-14.
36. Khaleghi A, Mohammadi MR, Zandifar A, et al. Epidemiology of psychiatric disorders in children and adolescents; in Tehran, 2017. *Asian journal of psychiatry* 2018; **37**: 146-53.
37. Kim-Cohen J, Caspi A, Moffitt TE, Harrington H, Milne BJ, Poulton R. Prior Juvenile Diagnoses in Adults With Mental Disorder: Developmental Follow-Back of a Prospective-Longitudinal Cohort. *Archives of General Psychiatry* 2003; **60**(7): 709-17.
38. Kinzl JF, Traweger C, Trefalt E, Mangweth B, Biebl W. Binge eating disorder in males: a population-based investigation. *Eating and weight disorders : EWD* 1999; **4**(4): 169-74.
39. La Maison C, Munhoz TN, Santos IS, Anselmi L, Barros FC, Matijasevich A. Prevalence and risk factors of psychiatric disorders in early adolescence: 2004 Pelotas (Brazil) birth cohort. *Social psychiatry and psychiatric epidemiology* 2018; **53**(7): 685-97.
40. Lähteenmäki S, Saarni S, Suokas J, et al. Prevalence and correlates of eating disorders among young adults in Finland. *Nordic journal of psychiatry* 2014; **68**(3): 196-203.
41. Ma QM, Li XP, Zhang HY, et al. Epidemiological Survey of Mental Disorder in Baoding. *Chin Gen Pract* 2008; **11**(6): 522-6.
42. Machado PP, Machado BC, Gonçalves S, Hoek HW. The prevalence of eating disorders not otherwise specified. *The International journal of eating disorders* 2007; **40**(3): 212-7.
43. Mangweth-Matzek B, Hoek HW, Rupp CI, et al. Prevalence of eating disorders in middle-aged women. *The International journal of eating disorders* 2014; **47**(3): 320-4.
44. Mangweth-Matzek B, Rupp CI, Hausmann A, et al. Never too old for eating disorders or body dissatisfaction: a community study of elderly women. *The International journal of eating disorders* 2006; **39**(7): 583-6.
45. Merikangas KR, He J-P, Brody D, Fisher PW, Bourdon K, Koretz DS. Prevalence and Treatment of Mental Disorders Among US Children in the 2001–2004 NHANES. *Pediatrics* 2010; **125**(1): 75-81.
46. Michel C, Schnyder N, Schmidt SJ, Groth N, Schimmelmann BG, Schultze-Lutter F. Functioning mediates help-seeking for mental problems in the general population. *European Psychiatry* 2018; **54**: 1-9.
47. Mohammadi MR, Ahmadi N, Kamali K, Khaleghi A, Ahmadi A. Epidemiology of Psychiatric Disorders in Iranian Children and Adolescents (IRCAP) and Its Relationship with Social Capital, Life Style and Parents' Personality Disorders: Study Protocol. *Iranian journal of psychiatry* 2017; **12**(1): 66-72.
48. Mohler-Kuo M, Schnyder U, Dermota P, Wei W, Milos G. The prevalence, correlates, and help-seeking of eating disorders in Switzerland. *Psychological medicine* 2016; **46**(13): 2749-58.
49. Mousa TY, Al-Domi HA, Mashal RH, Jibril MA. Eating disturbances among adolescent schoolgirls in Jordan. *Appetite* 2010; **54**(1): 196-201.
50. Qu Y, Jiang H, Zhang N, Wang D, Guo L. Prevalence of mental disorders in 6-16-year-old students in Sichuan province, China. *International journal of environmental research and public health* 2015; **12**(5): 5090-107.
51. Rosenvinge JH, Sundgot Borgen J, Börresen R. The prevalence and psychological correlates of anorexia nervosa, bulimia nervosa and binge eating among 15-year-old students: a controlled epidemiological study. *European Eating Disorders Review* 1999; **7**(5): 382-91.

52. Ruiz-Lazaro PM, Alonso JP, Comet P, Lobo A, Velilla M. Prevalence of Eating Disorders in Spain: A Survey on a Representative Sample of Adolescents.
2005, Swain, Pamela I. (2005). Trends in eating disorders research. (pp. 85-108). In: PL S, ed. Trends in Eating disorders research. Hauppauge, NY, US: Nova Biomedical Books; 2005: 85-108.
53. Salum GA, Gadelha A, Pan PM, et al. High risk cohort study for psychiatric disorders in childhood: rationale, design, methods and preliminary results. *International journal of methods in psychiatric research* 2015; **24**(1): 58-73.
54. Sancho C, Arija MV, Canals J. Personality in non-clinical adolescents with eating disorders. *European eating disorders review : the journal of the Eating Disorders Association* 2008; **16**(2): 133-8.
55. Semız M, Kavakcı Ö, Yağız A, Yontar G, Kuğu N. [The prevalence of eating disorders and comorbid psychiatric disorders in the Sivas Province]. *Turk psikiyatri dergisi = Turkish journal of psychiatry* 2013; **24**(3): 149-57.
56. Smink FRE, van Hoeken D, Oldehinkel AJ, Hoek HW. Prevalence and severity of DSM-5 eating disorders in a community cohort of adolescents. *International Journal of Eating Disorders* 2014; **47**(6): 610-9.
57. Solmi F, Hotopf M, Hatch SL, Treasure J, Micali N. Eating disorders in a multi-ethnic inner-city UK sample: prevalence, comorbidity and service use. *Social psychiatry and psychiatric epidemiology* 2016; **51**(3): 369-81.
58. Swanson SA, Crow SJ, Le Grange D, Swendsen J, Merikangas KR. Prevalence and correlates of eating disorders in adolescents. Results from the national comorbidity survey replication adolescent supplement. *Arch Gen Psychiatry* 2011; **68**(7): 714-23.
59. Szumska I, Túry F, Csoboth CT, Réthelyi J, Purebl G, Hajnal Á. The prevalence of eating disorders and weight-control methods among young women: a Hungarian representative study. *European Eating Disorders Review* 2005; **13**(4): 278-84.
60. Wagner G, Zeiler M, Waldherr K, et al. Mental health problems in Austrian adolescents: a nationwide, two-stage epidemiological study applying DSM-5 criteria. *European child & adolescent psychiatry* 2017; **26**(12): 1483-99.
61. Wells JE, Oakley Browne MA, Scott KM, McGee MA, Baxter J, Kokaua J. Prevalence, Interference with Life and Severity of 12 Month DSM-IV Disorders in Te Rau Hinengaro: The New Zealand Mental Health Survey. *Australian & New Zealand Journal of Psychiatry* 2006; **40**(10): 845-54.
62. West P, Sweeting H, Der G, Barton J, Lucas C. Voice-DISC identified DSM-IV disorders among 15-year-olds in the west of Scotland. *Journal of the American Academy of Child and Adolescent Psychiatry* 2003; **42**(8): 941-9.
63. Wittchen HU, Nelson CB, Lachner G. Prevalence of mental disorders and psychosocial impairments in adolescents and young adults. *Psychological medicine* 1998; **28**(1): 109-26.
